# Muscle Contraction Regulates BDNF/TrkB Signaling to Modulate Synaptic Function through Presynaptic cPKCα and cPKCβI

**DOI:** 10.3389/fnmol.2017.00147

**Published:** 2017-05-18

**Authors:** Erica Hurtado, Víctor Cilleros, Laura Nadal, Anna Simó, Teresa Obis, Neus Garcia, Manel M. Santafé, Marta Tomàs, Katherine Halievski, Cynthia L. Jordan, Maria A. Lanuza, Josep Tomàs

**Affiliations:** ^1^Unitat d’Histologia i Neurobiologia (UHNEUROB), Facultat de Medicina i Ciències de la Salut, Universitat Rovira i VirgiliReus, Spain; ^2^Neuroscience Program, Michigan State UniversityMichigan, MI, United States

**Keywords:** Bdnf-TrkB signaling, neuromuscular junction, PKC, muscle contraction, neurotransmission

## Abstract

The neurotrophin brain-derived neurotrophic factor (BDNF) acts via tropomyosin-related kinase B receptor (TrkB) to regulate synapse maintenance and function in the neuromuscular system. The potentiation of acetylcholine (ACh) release by BDNF requires TrkB phosphorylation and Protein Kinase C (PKC) activation. BDNF is secreted in an activity-dependent manner but it is not known if pre- and/or postsynaptic activities enhance BDNF expression *in vivo* at the neuromuscular junction (NMJ). Here, we investigated whether nerve and muscle cell activities regulate presynaptic conventional PKC (cPKCα and βI) via BDNF/TrkB signaling to modulate synaptic strength at the NMJ. To differentiate the effects of presynaptic activity from that of muscle contraction, we stimulated the phrenic nerve of rat diaphragms (1 Hz, 30 min) with or without contraction (abolished by μ-conotoxin GIIIB). Then, we performed ELISA, Western blotting, qRT-PCR, immunofluorescence and electrophysiological techniques. We found that nerve-induced muscle contraction: (1) increases the levels of mature BDNF protein without affecting pro-BDNF protein or BDNF mRNA levels; (2) downregulates TrkB.T1 without affecting TrkB.FL or p75 neurotrophin receptor (p75) levels; (3) increases presynaptic cPKCα and cPKCβI protein level through TrkB signaling; and (4) enhances phosphorylation of cPKCα and cPKCβI. Furthermore, we demonstrate that cPKCβI, which is exclusively located in the motor nerve terminals, increases activity-induced acetylcholine release. Together, these results show that nerve-induced muscle contraction is a key regulator of BDNF/TrkB signaling pathway, retrogradely activating presynaptic cPKC isoforms (in particular cPKCβI) to modulate synaptic function. These results indicate that a decrease in neuromuscular activity, as occurs in several neuromuscular disorders, could affect the BDNF/TrkB/PKC pathway that links pre- and postsynaptic activity to maintain neuromuscular function.

## Introduction

Nerves and skeletal muscles interact via two modes of communication: electrical activity and neurotrophic regulation (Baldwin et al., 2013; Cisterna et al., 2014). Nerve impulses generated in the central nervous system (CNS) trigger muscle contraction via electromechanical coupling. On the other hand, neurotrophic control acts via the release of neurotrophic factors, including the neurotrophins, and regulates the development, differentiation, survival and function of the nerve terminal (Wang et al., 1995; Mantilla et al., 2004). One of the most studied neurotrophins is brain-derived neurotrophic factor (BDNF; Hofer and Barde, 1988; Barde, 1990; Bibel and Barde, 2000). BDNF is initially synthesized as a precursor (proBDNF), which is cleaved to a mature isoform (mBDNF) via intracellular or extracellular proteases. The two isoforms induce different and even opposite functions by binding preferentially to the low-affinity nerve growth factor receptor (p75) or the tropomyosin-related kinase B receptor (TrkB; Lu, 2003; Hempstead, 2006; Kermani and Hempstead, 2007; Yang et al., 2009; Je et al., 2013). In addition, alternative splicing of TrkB mRNA gives rise to a full-length TrkB isoform (TrkB.FL) and two truncated TrkB isoforms T1 and T2 (TrkB.T1 and TrkB.T2), which lack part of the intracellular kinase domain (Middlemas et al., 1991; Reichardt, 2006). TrkB.T1 is the main truncated isoform in the skeletal muscle, being TrkB.T2 a variant more predominant in the brain tissue (Stoilov et al., 2002). Evidence suggests that heterodimers of TrkB.FL with the truncated isoforms inhibit trans-autophosphorylation of TrkB.FL, reduce BDNF signaling or even may signal independently (Eide et al., 1996; Baxter et al., 1997; Rose et al., 2003; Dorsey et al., 2012; Wong and Garner, 2012).

Increasing evidence suggests that exercise training benefits CNS health, including the improvement of synaptic function (van Praag et al., 1999). BDNF seems to play a key role in mediating the benefits of exercise (Neeper et al., 1995; Vaynman et al., 2006; Gomez-Pinilla et al., 2008; Zoladz and Pilc, 2010; Gomez-Pinilla and Hillman, 2013). In particular, BDNF is secreted in an activity-dependent manner (Lu, 2003) and its expression in rodent spinal cord and skeletal muscle increases after exercise (Gómez-Pinilla et al., 2001, 2002; Cuppini et al., 2007; Gomez-Pinilla et al., 2012). Likewise, basal levels of neuromuscular activity are required to maintain normal levels of BDNF in the neuromuscular system (Gómez-Pinilla et al., 2002). Recently, it was shown that cultured myotubes release BDNF when stimulated to contract, suggesting a postsynaptic origin of this neurotrophin (Matthews et al., 2009). Unfortunately, whether skeletal muscles *in vivo* increase their production and/or release of BDNF by synaptic activity, muscle contraction or some combination of the two, remains unclear. Furthermore, exogenous BDNF increases evoked acetylcholine (ACh) release at the neuromuscular junction (NMJ) and the TrkB receptor is normally coupled to this process (Knipper et al., 1994; Mantilla et al., 2004; Garcia et al., 2010; Santafé et al., 2014). Together, this and other findings support the idea that neuromuscular activity promotes BDNF/TrkB retrograde signaling to regulate neuromuscular function (Kulakowski et al., 2011; Dorsey et al., 2012), an idea we now test.

The potentiation of presynaptic vesicle released by BDNF requires TrkB phosphorylation and phospholipase C (PLC) activation (Middlemas et al., 1994; Kleiman et al., 2000). In turn, PLCγ activates Protein Kinase C (PKC) which interacts with TrkB to modulate neurotransmission at the NMJ (West et al., 1991; Numann et al., 1994; Byrne and Kandel, 1996; Catterall, 1999; Santafé et al., 2005, 2006, 2014). In the NMJ, synaptic activity depends on the influx of calcium, and presynaptic calcium-dependent PKC (cPKC) isoforms have been shown to modulate neurotransmission (Santafé et al., 2005, 2006; Besalduch et al., 2010). However, which cPKC isoforms are involved in ACh release remains unknown. The cPKCβI and cPKCα isoforms are good candidates because of their presynaptic location, with PKCβI being present exclusively in the nerve terminal of the NMJ (Besalduch et al., 2010). Recently, we have shown that muscle contraction *per se* enhances the levels of presynaptic PKC isoforms (α, βI and ε; Besalduch et al., 2010; Obis et al., 2015a). This suggests that a retrograde signal induced by muscle contractile activity can regulate presynaptic PKC isoforms.

Here, we investigated the hypothesis that nerve-induced muscle activity regulates BDNF/TrkB signaling pathway to modulate synaptic function via activation of presynaptic cPKC isoforms.

## Materials and Methods

### Animals

Diaphragm and levator auris longus (LAL) muscles were obtained from Sprague-Dawley rats (30–40 days; Criffa, Barcelona, Spain). The animals were cared for in accordance with the guidelines of the European Community Council Directive of 24 November 1986 (86/609/EEC) for the humane treatment of laboratory animals. All the procedures realized were reviewed and approved by the Animal Research Committee of the Universitat Rovira i Virgili (URV; Reference number: 0289). At least five independent animals (*n* > 5) were used to evaluate the following techniques.

### Antibodies

Primary antibodies used for Western blot were rabbit anti-BDNF (Cat# sc-20981), rabbit anti-PKCα (Cat# sc-208), rabbit anti-PKCβI (Cat# sc-209), rabbit anti-TrkB (Cat# sc-8316) and goat anti-glyceraldehyde 3-phosphate dehydrogenase (GAPDH; Cat# sc-20358) polyclonal antibodies, purchased from Santa Cruz Biotechnology. Rabbit anti-pPKCα (ser657; Cat# 07-790), goat anti-p75 (Cat# AB1554), rabbit anti-neurotrophin-4 (NT-4; Cat# AB1781SP) and rabbit anti-pTrkB (tyr816; Cat# AB1381) antibodies were purchased from Merck Millipore. Rabbit anti-pPKCβI (thr 641; Cat# ab75657) polyclonal antibody was purchased from Abcam. The secondary antibodies used were donkey anti-rabbit conjugated to horseradish peroxidase (HRP) from Jackson Immunoresearch Labs (Cat# 711-035-152) and rabbit anti-goat conjugated to HRP from Molecular probes (Cat# R21459). Immunohistochemistry was performed with antibodies that are commonly used as markers to differentially detect the components of the NMJ (syntaxin, neurofilament-200 and S100): mouse anti-syntaxin (Cat# S0664) and mouse anti-neurofilament-200 (Cat# N2912) monoclonal antibodies were purchased from Sigma. Mouse anti-S100 monoclonal antibody (Cat# AM10036FC-N) was from Acris Antibodies. Rabbit anti-PKCβI polyclonal antibody was purchased from Santa Cruz Biotechnology (Cat# sc-209). The secondary antibodies used were donkey anti-rabbit or anti-mouse conjugated to Alexa Fluor 488 and Alexa Fluor 647 from Molecular Probes (Eugene, OR, USA; Cat# A21206; Cat# A21202; Cat# A-31573; Cat# A-31571). Postsynaptic AChRs were detected with α-bungarotoxin (α-BTX) conjugated to tetramethylrhodamine (TRITC) from Molecular Probes (Eugene, OR, USA; Cat# T1175). As a control, primary antibodies were omitted from some muscles during the immunohistochemical and Western blot procedures. These control muscles never exhibited positive staining or revealed bands of the appropriate molecular weight with the respective procedures. In double-staining protocols, omitting either one of the two primary antibodies completely abolished the corresponding staining and there was no cross-reaction with the other primary antibody. Pretreatment of a primary antibody with the appropriate blocking peptide (between three- and eight-fold by weight) in skeletal muscle tissue prevented immunolabelling.

### Reagents

In presynaptic stimulation treatments and electrophysiological experiments, muscle contraction was blocked using μ-conotoxin GIIIB (μ-CgTx-GIIIB, Alomone Labs Ltd, Jerusalem, Israel). This toxin selectively inhibits sarcolemmal voltage-dependent sodium channels (VDSCs) without affecting synaptic ACh release (Favreau et al., 1999). It was supplied as lyophilized powder of >99% purity. The working concentration was 1.5 μM in Ringer’s solution (see below).

The anti-TrkB antibody clone 47/TrkB (BD Transduction Laboratories Cat# 610101) was used for TrkB inhibition assays. The working solution was 10 μg/ml. For BDNF exogenous incubations we used h-BDNF (Alomone Labs; Cat# B-250) 10 mM.

Phosphatase inhibition experiments were performed using a phosphatase inhibitor cocktail from Sigma-Aldrich (St. Louis, MO, USA) in a 100-fold dilution.

To block cPKCβI activity we used the specific translocation inhibitor peptide βIV5–3 at 10 μM (kindly provided by Dr. Mochly-Rosen from Stanford University). It is derived from the V5 domain of cPKCβI and binds to the anchoring protein RACK (receptors for activated C-kinase), disrupting the interaction between cPKCβI and its specific βI-RACK. This inhibits its translocation to the membrane and its activation.

### Presynaptic Electrical Stimulation of Muscles

Diaphragm muscle—a typical model to study the development and function of the NMJ (Yang et al., 2001; Li et al., 2008; Wu et al., 2010)—was excised together with its nerve supply and dissected into two hemidiaphragms. One hemidiaphragm underwent a treatment and the other one was used as its control. The experimental design of the treatments is shown in Table 1. The protocol of electrical stimulation followed that of Besalduch et al. (2010) and Obis et al. (2015a). Briefly, each hemidiaphragm muscle with the phrenic nerve was placed in oxygenated Ringer solution (in nM: NaCl 137, KCl 5, CaCl_2_ 2, MgSO_4_ 1, NaH_2_PO_4_ 1, NaHCO_3_ 12 and glucose 12.1 mM) continuously bubbled with 95% O_2_/5% CO_2_ at room temperature. The phrenic nerve was stimulated *ex vivo* at 1 Hz by an A-M Systems 2100 isolated pulse generator (A-M System, Carlsborg, WA, USA). The frequency of 1 Hz allows the maintenance of different tonic functions (e.g., PKC activation) without promoting synaptic plasticity (e.g., facilitation). To study separately the effect of synaptic transmission from the effect of the muscle cell contraction, we performed experiments in which contractions were prevented using μ-CgTx-GIIIB or not. Visible contractions of the diaphragm muscle served to verify successful nerve stimulation. Three main experiments were performed to discern the effects of synaptic activity from that of muscle activity (Table 1). Each experiment involved a specific treatment and its control. In Experiment #1, synaptic activity was assessed comparing presynaptically stimulated muscles blocked by μ-CgTx-GIIIB with non-stimulated muscles also incubated with μ-CgTx-GIIIB to control for nonspecific effects of the blocker. In Experiment #2, muscle contraction *per se* was determined comparing stimulated/contracting muscles with stimulated muscles for which contraction was blocked. In Experiment #3, the effect of complete synaptic activity with resulting muscle contraction was assessed comparing stimulated/contracting muscles with non-stimulated muscles, without exposure to μ-CgTx-GIIIB. Phrenic nerves were stimulated for 30 min unless otherwise noted. In all the cases, a minimum of five animals were used.

**Table 1:** **Summary of the electrical stimulation experiments applied to extracted rat diaphragms**.

Experiment	Control treatment		Treatment		Final outcome	
	**No stimulation, blocked contraction**		**Stimulation, blocked contraction**			
**#1 Presynaptic stimulation**	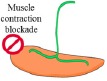	Hemidiaphragm extraction. μ-conotoxin GIIIB preincubation. Incubation in Ringer solution without stimulation.	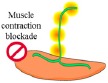	Hemidiaphragm extraction. μ-conotoxin GIIIB preincubation. Phrenic nerve stimulation with contraction blocked.	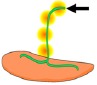	**Effect of presynaptic stimulation**
	**Stimulation, blocked contraction**		**Stimulation, contraction**			
**#2 Contraction**	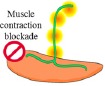	Hemidiaphragm extraction. μ-conotoxin GIIIB preincubation. Phrenic nerve stimulation with contraction blocked.	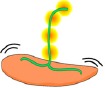	Hemidiaphragm extraction. Preincubation in Ringer solution. Phrenic nerve stimulation with contraction.	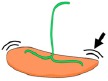	**Effect of muscle contraction**	
	**No stimulation, not blocked contraction**		**Stimulation, contraction**			
**#3 Presynaptic stimulation with contraction**	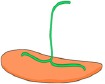	Hemidiaphragm extraction. Incubation in Ringer solution without stimulation.	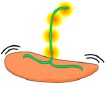	Hemidiaphragm extraction. Phrenic nerve stimulation with contraction.	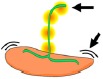	**Effect of presynaptic stimulation with contraction**.

*Three main experiments were designed in order to separately study the effect of the presynaptic stimulation (and synaptic transmission), the effect of muscle cell contraction and the effect of presynaptic stimulation with muscle contraction. Each diaphragm was divided into two hemidiaphragms, one underwent a treatment and the other served as its particular control*.

#### BDNF ELISA Assay

Diaphragm muscles were dissected, frozen in liquid nitrogen and homogenized using a high-speed homogenizer (overhead stirrer, VWR International, Clarksburg, MD, USA) in ice-cold lysis buffer (in mM: NaCl 150, Tris-HCl (pH 7.4) 50, EDTA 1, NaF 50, PMSF 1, sodium orthovanadate 1; NP-40 1%, Triton X-100 0.1% and protease inhibitor cocktail 1% (Sigma-Aldrich, St. Louis, MO, USA). Protein lysates were obtained collecting supernatants after removing insoluble materials by centrifugation at 1000 *g* for 10 min at 4°C and then at 15,000 *g* for 20 min at 4°C.

Protein concentrations were determined by DC protein assay (Bio-Rad, Hercules, CA, USA). The BDNF Emax ImmunoAssay System (Promega Cat# G7610) was used to measure the amount of total BDNF (pro- and mature) in each sample following standard protocols. Plates were coated with a specific anti-BDNF monoclonal antibody and then washed once using tris-buffered saline, 0.1% Tween 20 (TSBT). Plates were blocked using 200 μL Promega 1× Block and Sample buffer. After washing, diaphragm samples and BDNF protein standards (0–500 pg BDNF protein) were added in triplicate to the plates and incubated for 2 h at room temperature. Plates were washed five times and anti-human BDNF polyclonal antibody was added to each well. Plates were washed again five times using TBST wash buffer. The sandwich was completed by adding anti-IgY HRP conjugate. Finally, plates were developed using stabilized chromogen (tetramethylbenzidine) and the reaction was stopped using 1 N HCl. Absorbance was read at 450 nm using a Sunrise Tecan A-5082 microplate reader and data was analyzed with Magellan software (Tecan Group Ltd.). Afterwards, the amount of detected BDNF was normalized to a standard curve and to the total protein content determined with the colorimetric assay (Bio-Rad).

#### Western Blot

Diaphragm muscles with the phrenic nerve were dissected, frozen in liquid nitrogen, and stored at −80°C before use. Homogenization, lysate obtainment and determination of protein concentration were performed with the same protocols and solutions described previously for BDNF ELISA assay. The linear and quantitative dynamic range for each target protein and the appropriate dilutions of samples were determined for accurate and normalized quantitation of densitometric analysis.

To isolate the membrane and cytosolic fractions, diaphragm muscles were dissected and homogenized in ice-cold lysis buffer without detergents (in mM: NaCl 150, Tris-HCl (pH 7.4) 50, EDTA 1, NaF 50, PMSF 1 and sodium orthovanadate 1 and protease inhibitor cocktail (1/100). The homogenized samples were cleared with a centrifugation at 1000 *g* at 4°C for 15 min, and the resulting supernatant was further centrifuged at 130,000 *g* at 4°C for 1 h. The supernatant corresponded to the cytosolic fraction and the pellet, to the membrane fraction. The latter was resuspended in lysis buffer (in mM: NaCl 150, Tris-HCl (pH 7.4) 50, EDTA 1, NaF 50, PMSF 1, sodium orthovanadate 1; NP-40 1%, Triton X-100 0.1% and protease inhibitor cocktail 1% (Sigma-Aldrich, St. Louis, MO, USA)). Fractionation was assessed by blotting of GAPDH, a specific cytosolic protein. GADPH immunoreactivity was never observed in the membrane fraction. Protein concentrations were determined as previously described.

Protein samples of 15 μg or 30 μg were separated by 8% or 15% SDS-polyacrylamide electrophoresis and electrotransferred to polyvinylidene difluoride (PVDF) membranes (Hybond^TM^-P; Amersham, GE Healthcare). The membranes were blocked in TBST containing 5% (W/V) nonfat dry milk or 5% bovine serum albumin (BSA) and probed with the primary antibody overnight at 4°C. The membranes were incubated with the secondary antibody and visualized by chemiluminescence with the ECL kit (Amersham Life Science, Arlington Heights, IL, USA).

The blots were visualized with the ChemiDoc XRS+Imaging System (Bio-Rad, Hercules, CA, USA). The densitometry of the resultant bands was analyzed with the ImageJ software (ImageJ). The integrated optical density of the bands was normalized with respect to: (1) the background values; and to (2) the total protein transferred on PVDF membranes, measured by total protein analysis (Sypro Ruby protein blot stain, Bio-rad; Aldridge et al., 2008). In some cases, β-actin blotting and total protein staining were used as a loading controls resulting in the same normalization values. Specific phosphorylation was determined as the ratio of phosphorylated protein to total protein content. The relative variations between the experimental samples and the control samples were calculated from the same membrane image. The data were taken from densitometry measurements made in at least five separate experiments, plotted against controls. Data are mean values ± SEM. Statistical significance of the differences between groups was evaluated under the Wilcoxon test or the Student’s *t*-test and the normality of the distributions was tested with the Kolmogorov–Smirnov test. The criterion for statistical significance was *p* < 0.05 vs. the control (*).

#### Gene Expression Analysis

Gene expression was analyzed via qRT-PCR in separate cohorts of rats from the three stimulation treatments (see Table 1) targeting four BDNF exons (IV, VI, VIII and IX), to determine whether changes in BDNF mRNA levels might underlie the changes in BDNF protein and whether this occurs preferentially at known activity-dependent exons.

Each rat provided one hemidiaphragm for the control group and the other hemidiaphragm for the experimental group (*n* = 6–7 hemidiaphragms per group), as described above for protein analysis. After 30 min of treatment (Table 1), tissue samples were frozen in RNase-free tubes in liquid nitrogen, and held at −80°C until processed. Instruments used for dissection were cleaned with RNaseZap (Sigma-Aldrich) between animal harvests.

RNeasy Fibrous Tissue Mini Kit (Qiagen) was used to extract RNA from muscle samples. Tissue was mechanically homogenized with a PRO200 homogenizer (Pro Scientific). Following extraction, RNA was quantified on a spectrophotometer (Beckman DU 530) by measuring 260 nm absorbance values. Extracted RNA was then reverse transcribed using the High Capacity cDNA Reverse Transcription Kit (Applied Biosystems) with the following thermocycle: 25°C for 10 min, 37°C for 2 h and 85°C for 5 min. Each qRT-PCR sample included 2.5 ng of cDNA, primers, and Power SYBR Green PCR Master Mix (Applied Biosystems). Thermocycle for the quantitative step on the ABI PRISM 7000 Sequence Detection System was as follows: 50°C for 2 min, 95°C for 10 min, and 40 cycles of 95°C for 15 s and 60°C for 1 min. A dissociation curve was determined for each well to confirm that only one product was amplified. Each sample was run in triplicate. Samples without reverse transcriptase during the cDNA conversion were also assessed to ensure that there was no DNA contamination. The reference gene was 18S (400 nM primers: GGAGCCTGCGGCTTAATTTG and CCACCCACGGAATCGAGAAA). In each experiment, we confirmed that levels of the reference gene were equivalent between treatment groups. Transcripts of four BDNF exons were quantified: IV (activity-dependent; 200 nM primers: ACTGAAGGCGTGCGAGTATT and GGTGGCCGATATGTACTCCTG), VI (activity-dependent; 200 nM primers: TCGCACGGTCCCCATTG and GGTCTCATCAAAGCCTGCCA), VIII (activity-independent; 400 nM primers: AAACAAATTCTGCCAGTCCTGC and TTGGATAACTGCTCTGCTCCG) and IX (total transcripts; 200 nM primers: GTCAAGTGCCTTTGGAGCCT and TGTTTGCGGCATCCAGGTAA). Optimal concentrations and amplification efficiencies were calculated for each primer set.

Relative Expression Software Tool (REST) was used to assess statistical significance and fold change of genes (Pfaffl et al., 2002). Specifically, this software uses the non-parametric Pair-Wise Fixed Reallocation Randomization Test to account for amplification efficiencies when determining fold change. It measures relative expression of a target gene (BDNF transcripts IV, VI, VIII and total IX) between a control and sample group following the normalization of the target gene to a reference gene (18S). The criterion for statistical significance was *p* < 0.05 (*).

#### Immunohistochemistry and Confocal Microscopy

Diaphragm and LAL muscles were processed by immunohistochemistry to detect and localize the cPKCβI isoform at the NMJ. LAL muscle permits a better imaging and analysis of NMJs within the muscle. Whole muscle mounts were fixed with 4% paraformaldehyde for 30 min. After fixation, the muscles were rinsed with PBS and incubated in 0.1 M glycine in phosphate buffer saline (PBS). The muscles were permeabilized with 0.5% Triton X-100 in PBS, and nonspecific binding was blocked with 4% BSA. Then, muscles were incubated overnight at 4°C in mixtures of three primary antibodies raised in different species (anti cPKCβI isoform; anti-syntaxin and anti-neurofilament-200 to label the axon terminal; anti-S100 to label Schwann cells) and then rinsed. The muscles were then incubated for 4 h at room temperature in a mixture of appropriate secondary antibodies. Acetylcholine receptors (AChRs) were detected with α-BTX conjugated with TRITC. At least three muscles were used as negative controls as described above. No crossover was detected between antibodies. For improved localization of the cPKCβI isoform at the NMJ, the muscles were processed to obtain semithin cross-sections from whole-mount multiple-immunofluorescent stained muscles. This method provided a simple and sensitive procedure for analyzing the cellular distribution of molecules at the NMJ (Lanuza et al., 2007). Immunolabeled NMJs from the whole-mount muscles and the semithin cross-sections were viewed with a laser-scanning confocal microscope (Nikon TE2000-E). Special consideration was given to the possible contamination of one channel by another. In experiments involving negative controls, the photomultiplier tube gains and black levels were identical to those used for a labeled preparation made in parallel with the control preparations. At least 25 endplates per muscle were observed, and at least six muscles were studied. Images were assembled using Adobe Photoshop software (Adobe Systems, San Jose, CA, USA) and neither the contrast nor brightness were modified.

#### Electrophysiology

Diaphragm muscles were removed surgically and incubated in a Sylgard-Petri dish containing normal Ringer solution (in mM): NaCl 135, KCl 5, CaCl_2_ 2.5, MgSO_4_ 1, NaH_2_PO_4_ 1, NaHCO_3_ 15, glucose 11 continuously bubbled with 95% O_2_ and 5% CO_2_. Temperature and humidity were set to 26°C and 50%, respectively. Spontaneous miniature endplate potentials (MEPPs) and evoked EPPs (EPPs) were recorded intracellularly with conventional glass microelectrodes filled with 3 M KCl (resistance: 20–40 MW). Recording electrodes were connected to an amplifier (Tecktronics, AMS02), and a distant Ag-AgCl electrode connected to the bath solution via an agar bridge (agar 3.5% in 137 mM NaCl) was used as a reference. The signals were digitized (DIGIDATA 1322A Interface, Axon Instruments Inc., Union City, CA, USA), stored and computer-analyzed. The software Axoscope 9.0 (Axon Instruments Inc., Union City, CA, USA) was used for data acquisition and analysis. To prevent muscle contraction during EPP recordings, we used μ-CgTx-GIIIB (1, 5 μM). After a muscle fiber had been impaled, the phrenic nerve was continuously stimulated (70 stimuli, 1 Hz) using two platinum electrodes that were coupled to a pulse generator (CIBERTEC CS-20) linked to a stimulus isolation unit. Thus, in stimulated muscles, we recorded and measured control EPPs and then, we incubated the muscle in βIV_5–3_ inhibitor peptide for 1 h. The last 50 EPPs were recorded. We selected fibers with membrane potentials of no less than −70 mV and used only those results from preparations which did not deviate by more than 5 mV during the recording. The mean amplitude (mV) per fiber was calculated and corrected for non-linear summation (EPPs were usually more than 4 mV; McLachlan and Martin, 1981) assuming a membrane potential of –80 mV. We studied a minimum of 15 fibers per muscle and usually a minimum of five muscles in each type of experiment. The statistical software SPSS© v17.0 (SPSS, RRID:SCR_002865) was used to analyze the results. Values are expressed as means ± SEM. Only one hemidiaphragm was used from each animal for a given experiment. We used the two-tailed Welch’s *t*-test (for unpaired values and variances were not assumed to be equal). Differences were considered significant at *p* < 0.05 (*).

### Results

To determine the relationship between neuromuscular activity and neurotrophic control, we developed an *in vivo* experimental system in which we can distinguish the effects of synaptic activity from that of muscle contraction (see Table 1). Synaptic activity includes normal presynaptic stimulus, synaptic transmission and endplate potential generation due to ACh signaling. Muscle contraction includes membrane depolarization of the muscle fiber involving voltage-dependent sodium channels and the resulting myofiber contraction. Thus, the effects of synaptic activity were determined by comparing muscles that had contraction blocked (with μ-CgTx-GIIIB), but the nerve was stimulated in one case but not the other (referred to as the *Stimulation* condition in the figures). The effects of muscle contraction were determined by comparing muscles which were both stimulated via their nerves, but in one case muscle contraction was blocked (referred to as the *Contraction* condition in the figures). Finally, presynaptic *Stimulation with Contraction* treatment comprises the effects of synaptic activity and muscle contraction, showing complete neuromuscular activity.

#### Both Synaptic Activity and Muscle Contraction Enhance BDNF Protein Levels in the Skeletal Muscle

**Figure 1:**
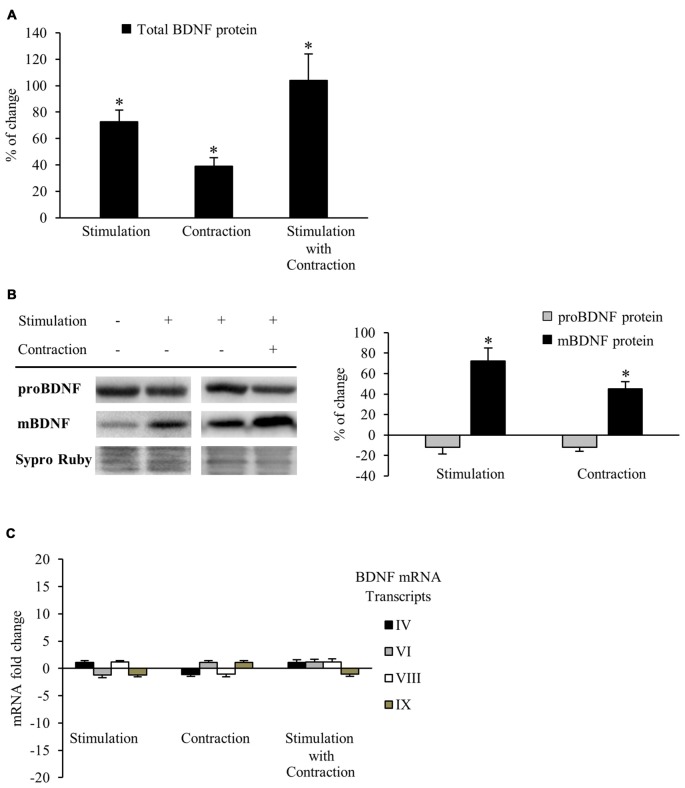
**Synaptic activity and muscle contraction increases brain-derived neurotrophic factor (BDNF) protein levels in the diaphragm muscle of rat. (A)** ELISA assessment of total BDNF in presynaptic stimulation treatment, contraction treatment and presynaptic stimulation with contraction treatment at 1 Hz stimulation for 30 min. Presynaptic stimulation has been simplified as Stimulation. Each column has been compared to its respective control (see Table 1). Data show that both presynaptic stimulation and muscle contraction enhanced BDNF levels. Specifically, presynaptic stimulation significantly increased BDNF protein levels but muscle contraction was able to increase them further. **(B)** Western blot bands and quantification of their optical density show that after stimulation protocols with or without muscle contraction (1 Hz stimulation for 30 min), proBDNF (32 kDa) levels remained the same and mBDNF (14 kDa) increased. **(C)** Quantitative real-time PCR did not show any difference in the expression of *bdnf* mRNA transcripts (IV and VI are activity-dependent, VIII is activity-independent, and IX represents total transcripts) after any stimulation protocol (1 Hz stimulation for 30 min). mRNA data are mean fold change ± SEM, protein data are mean percentage ± SEM, *p < 0.05. Abbreviations: mBDNF, mature BDNF; proBDNF, precursor BDNF.

Neuromuscular activity (e.g., through physical exercise) increases BDNF expression in the skeletal muscle, CNS and plasma (Gómez-Pinilla et al., 2001, 2002; Cuppini et al., 2007; Zoladz and Pilc, 2010; Gomez-Pinilla et al., 2012). However, it has been assumed that enhanced BDNF levels is caused by muscle activity *per se*, but this has not been directly shown. Therefore, our first objective was to determine whether synaptic activity and/or muscle contraction regulate BDNF protein levels in the skeletal muscle. Thus, we compare BDNF protein levels in muscles stimulated via the nerve (1 Hz) for which contraction was blocked or not. Each hemidiaphragm was compared to a corresponding control from the same animal (see Table 1). BDNF levels were measured using ELISA and results showed that nerve stimulation significantly elevated BDNF levels, either in the presence of contraction or when it was blocked. Synaptic activity raised total BDNF levels in muscle by 72.8% ± 8.6 (Figure 1A). Likewise, muscle contraction further increased total BDNF levels by 38.9% ± 6.5 (Figure 1A). In concordance, combined synaptic and muscle activity increased BDNF levels by 104.0% ± 19.9, indicating that the effects of synaptic vs. muscle activity had additive effects in increasing significantly the level of total BDNF protein in muscle. That nerve activity without muscle contraction potently increases BDNF expression in muscle is a novel finding. Altogether, these results indicate that both synaptic activity and muscle contraction enhance BDNF protein levels in skeletal muscle. Moreover, muscle contraction *per se* increases the BDNF levels above the rise produced by synaptic activity without contraction, significantly contributing to the total protein amount in skeletal muscle.

#### Synaptic Activity and Muscle Contraction each Increase Mature BDNF Protein Levels

Although results of the ELISA allowed us to quantify accurately the total BDNF protein levels in muscle for each experiment, this technique cannot tell us how the mBDNF and pro-BDNF are modulated by activity. Because pro-BDNF and mBDNF produce different, and even opposite, effects (Lu, 2003; Woo et al., 2005; Hempstead, 2006; Yang et al., 2009; Je et al., 2013), we used Western blotting to analyze how activity affected the level of mBDNF and pro-BDNF separately. We used an anti-BDNF antibody raised against a peptide sequence corresponding to the amino acids 130–247 of BDNF, a region present in both pro-BDNF (32 kDa) and mBDNF (14 kDa; Zheng et al., 2010). BDNF Western blotting (Figure 1B) shows that both presynaptic stimulation and muscle contraction each significantly increased mBDNF protein levels without affecting pro-BDNF. In particular, presynaptic stimulation induced a 72.5% ± 12.4 (*p* < 0.05) increase in mBDNF levels with respect to basal conditions whereas muscle contraction further increased mBDNF levels by 45.2% ± 7.5 (*p* < 0.05). Although the source of this increased BDNF is not clear, our results show that activity in either the synapse or the muscle increase mature BDNF without affecting pro-BDNF protein levels in skeletal muscle.

That pro-BDNF levels were maintained in the face of increases in mBDNF levels suggests that synthesis of this isoform is tightly regulated, otherwise, pro-BDNF levels would decrease after the upregulation of its cleavage into mature BDNF. Thus, we examined gene expression via qRT-PCR to determine whether BDNF mRNA levels were enhanced after the different stimulation conditions. Surprisingly, no difference was found in the expression of any of the four BDNF mRNA transcripts (IV activity-dependent, VI activity-dependent, VIII activity-independent and IX total transcripts) examined after any condition (Figure 1C). Therefore, neither presynaptic stimulation nor muscle contraction are able to significantly increase *bdnf* mRNA transcription in skeletal muscle under our activity stimulation treatments. These results suggest that the maintenance of pro-BDNF could be due to an increase in BDNF mRNA translation from mRNA pools in the cells.

Because both BDNF and NT4 bind TrkB receptors, we also tested whether synaptic activity with or without muscle contraction regulates NT4 protein level in the skeletal muscle. Results showed that NT4 protein levels did not change under these conditions (presynaptic stimulation: 2.46 ± 1.34, *p* > 0.05; muscle contraction: −7.23 ± 5.84, *p* > 0.05; presynaptic stimulation with contraction: −8.61 ± 5.34, *p* > 0.05), suggesting that activity-dependent enhancement in TrkB-signaling is likely mediated by BDNF.

#### Muscle Contraction Reduces TrkB.T1 Receptor without Affecting TrkB.FL or p75 Protein Levels

**Figure 2:**
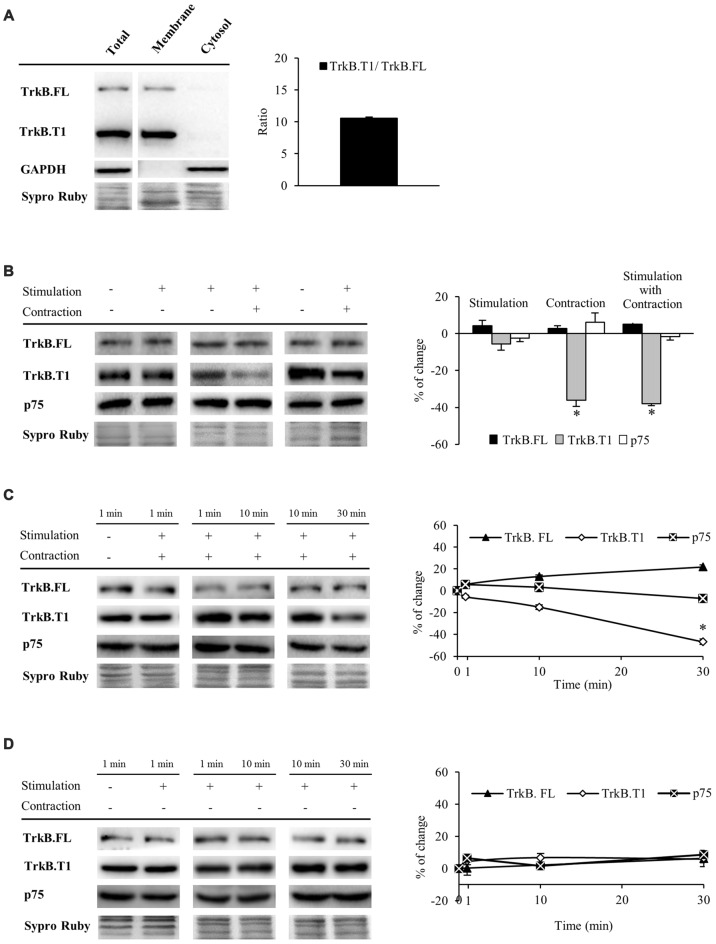
**Muscle contraction reduces TrkB.T1 isoform without affecting TrkB.FL or p75 protein levels**. Panels **(A–D)** show Western blot bands and their quantification.** (A)** TrkB.FL (145–150 kDa) and TrkB.T1 (95–100 kDa) were present in the total and membrane fractions and, in basal conditions, TrkB.T1 was the predominant form in the skeletal muscle. **(B)** TrkB.FL, TrkB.T1 and p75 receptors in presynaptic stimulation treatment, contraction treatment and presynaptic stimulation with contraction treatment at 1 Hz stimulation for 30 min. Presynaptic stimulation has been simplified as Stimulation. Each column has been compared to its respective control (see Table 1). TrkB.T1 was the only receptor modulated by activity, decreasing only when contraction was present. **(C)** TrkB.FL, TrkB.T1 and p75 receptors at 1, 10 and 30 min after presynaptic stimulation with contraction treatment. Each time-point has been compared to its previous time-point. Muscle contraction decreased TrkB.T1 between 10 min and 30 min, without altering TrkB.FL nor p75. **(D)** TrkB.FL, TrkB.T1 and p75 receptors at 1, 10 and 30 min after presynaptic stimulation treatment. Each time-point has been compared to its previous time-point. No receptor was altered without contraction. Data are mean percentage ± SEM, *p < 0.05. Abbreviations: TrkB.FL, Full Length isoform of TrkB; TrkB.T1, truncated isoform 1 of TrkB; p75, low-affinity nerve growth factor receptor.

Having established that muscle contraction driven by presynaptic stimulation enhances mBDNF protein levels, we next sought to analyze BDNF receptors that are expressed the most in the skeletal muscle (TrkB.FL, TrkB.T1 and p75). In order to study TrkB receptors, we used an anti-TrkB antibody raised against a peptide sequence corresponding to the amino acids 160–340 of TrkB, the extracellular domain shared by TrkB.FL (145–150 kDa) and TrkB.T1 (95–100 kDa; Figure 2A). Furthermore, we confirmed that TrkB.FL and TrkB.T1 bands were only found in the membrane fraction, being absent in the cytosol. In addition, no differences were found between the membrane and total fraction samples (Figure 2A). Therefore, to minimize the variability of sample processing, the following results were obtained from total fraction samples. Both receptors were expressed in skeletal muscle at basal conditions, TrkB.T1 being the predominant form (ratio T1/FL = 10.59 ± 0.41, Figure 2A). To determine if BDNF receptors are affected by activity, we analyzed how their protein levels were modulated under the stimulation treatments (Table 1 and Figure 2B). We observed that muscle contraction decreased the level of TrkB.T1 protein while presynaptic activity has no apparent effect (−36.13% ± 3.5, *p* < 0.05; −5.58% ± 3.45, *p* < 0.05, respectively). Moreover, none of the stimulation conditions affected TrkB.FL or p75 levels. Thus, the basal FL:T1 ratio was ~1:11 and contraction treatment elevated it to ~1:6. Furthermore, preincubation with exogenous BDNF with contraction yielded similar results (TrkB.T1: −24.18% ± 4.3, *p* < 0.05; neither TrkB.FL nor p75 were affected). Therefore, BDNF, which is enhanced by contraction, could contribute to the contraction-dependent decrease of TrkB.T1. However, presynaptic stimulation did not alter the level of any BDNF receptor indicating that muscle contraction *per se* is necessary for synaptic activity to reduce the amount of TrkB.T1 protein.

Since TrkB signaling is known to be quick (Klein et al., 1991; Suen et al., 1997; Takei et al., 1998; Aloyz et al., 1999), before analyzing pTrkB (TrkB phosphorylation), we first analyzed the time course of BDNF receptor levels at 1, 10 and 30 min of stimulation (Figures 2C,D). We found that under presynaptic stimulation with contraction, TrkB.FL and p75 protein levels are maintained whereas TrkB.T1 levels start to decrease within 10 min, becoming significant at 30 min of neuromuscular activity (Figure 2C). Presynaptic stimulation alone did not alter the levels of any receptor (Figure 2D).

Altogether, these results demonstrate that synaptic activity is not enough to modulate TrkB.FL, TrkB.T1 and p75 protein levels even though synaptic activity potently regulates mBDNF levels in the absence of muscle contraction. However, nerve-induced muscle contraction is necessary to downregulate TrkB.T1 levels.

#### Both Synaptic Activity and Muscle Contraction Modulate TrkB.FL Phosphorylation

**Figure 3:**
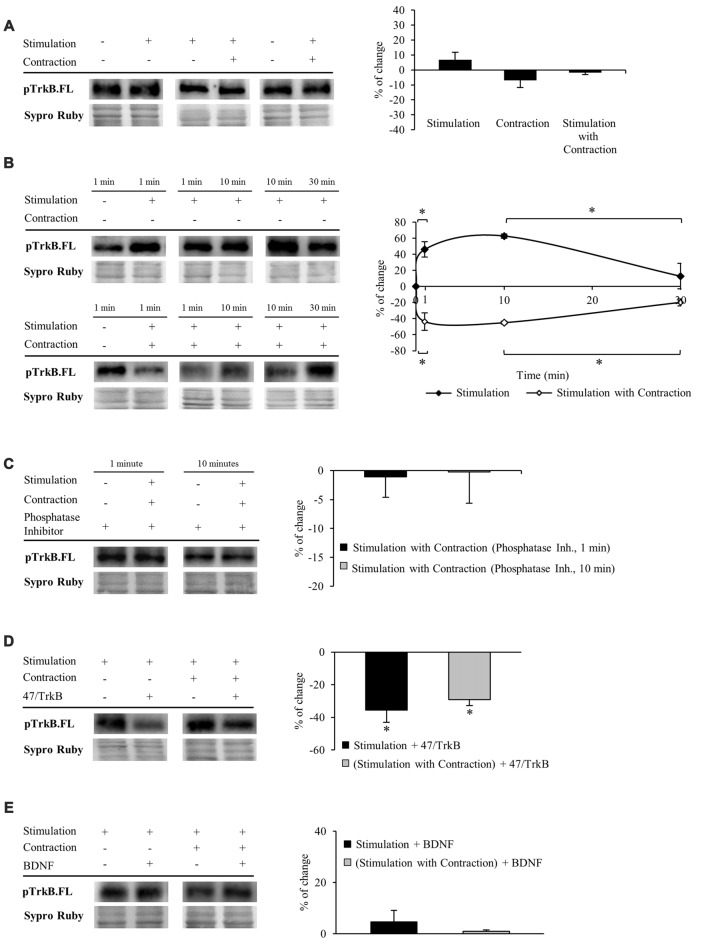
**Both synaptic activity and muscle contraction modulates TrkB phosphorylation at tyr816**. Panels **(A–E)** show Western blot bands and their quantification. **(A)** pTrkB.FL in presynaptic stimulation treatment, contraction treatment and presynaptic stimulation with contraction treatment at 1 Hz stimulation for 30 min. Presynaptic stimulation has been simplified as Stimulation. Each column has been compared to its respective control (see Table 1). pTrkB did not show any change after 30 min of increased activity. **(B)** pTrkB.FL at 1, 10 and 30 min under presynaptic stimulation and presynaptic stimulation with contraction treatments. Each time-point has been compared to its previous time-point. Presynaptic stimulation significantly increased pTrkB.FL at 1 min, an effect maintained after 10 min but declined significantly near baseline by 30 min of electrical stimulation. In the presence of muscle contraction, pTrkB.FL levels decreased after 1 min and increased back to baseline levels at 30 min of treatment. **(C)** Phosphatase inhibition prevented the decrease of pTrkB.FL under presynaptic stimulation with contraction at 1 and 10 min. **(D)** Effect of the sequestering antibody 47/TrkB (10 μg/ml) on pTrkB.FL under presynaptic stimulation and presynaptic stimulation with contraction treatments (1 Hz stimulation for 30 min). After both treatments, pTrkB.FL levels decreased. **(E)** Effect of exogenous BDNF (10 μM) on pTrkB.FL following 30 min under presynaptic stimulation and presynaptic stimulation with contraction treatments (1 Hz stimulation for 30 min). pTrkB.FL did not change under these conditions. Specific phosphorylation was determined as the ratio of phosphorylated protein to total protein content. Data are mean percentage ± SEM, *p < 0.05. Abbreviations: pTrkB.FL, phosphorylated TrkB.FL.

We next performed experiments to determine whether TrkB.FL shows enhanced phosphorylation. Specific phosphorylation was determined as the ratio of phosphorylated protein to total protein content (pTrkB.FL/TrkB.FL). We used an antibody which specifically recognizes the tyr816 phosphorylation of TrkB.FL, known to trigger the PLCγ signaling pathway that activates PKC. Unexpectedly, we did not detect any change in TrkB.FL phosphorylation in muscle after 30 min of nerve stimulation, regardless of whether the muscle contracted or not (Figure 3A).

This result led us to think that TrkB.FL phosphorylation may be quick and transient. Therefore, we analyzed pTrkB.FL at shorter times of stimulation with and without muscle contraction (1 min and 10 min, 1 Hz). Presynaptic stimulation for 1 min without muscle contraction significantly increased TrkB.FL phosphorylation (46.06% ± 13.36; Figure 3B, top), an effect maintained after 10 min of stimulation but declined back to near baseline by 30 min of electrical stimulation. These results suggest that activation of TrkB.FL induced by synaptic activity is very fast and only lasts for a short period of time. Surprisingly, presynaptic stimulation with muscle contraction significantly decreased pTrkB.FL after 1 min (−43.55% ± −15.84; Figure 3B, bottom). This reduction in TrkB phosphorylation was also evident at 10 min but returned to baseline levels after 30 min of neuromuscular activity. Interestingly, even shorter periods of presynaptic stimulation with contraction (10 s and 30 s) also significantly decreased pTrkB.FL levels (10 s: −33.49 ± 2.06, *p* < 0.05; 30 s: −41.63 ± 6.49, *p* < 0.05). These results indicate that while synaptic activity without muscle contraction positively regulates TrkB.FL phosphorylation, muscle contraction has the opposite effect, negatively regulating phosphorylation of TrkB.FL on the tyr816 very rapidly, suggesting a quick sequence of activation-phosphorylation-dephosphorylation for TrkB.FL. Because several phosphatases are involved in regulating TrkB signaling (Rusanescu et al., 2005; Ambjørn et al., 2013; Gatto et al., 2013; Ozek et al., 2014), we analyzed whether this decrease of the pTrkB.FL at short times (1 and 10 min) could be prevented by inhibiting phosphatases. Preincubation with a cocktail of phosphatase inhibitors prevented the decrease of the pTrkB.FL (1 min: −1.07 ± 3.52, *p* > 0.05; 10 min: −0.25 ± 5.37, *p* > 0.05; Figure 3C). This indicates that phosphatases regulate TrkB.FL phosphorylation of the PLCγ site when muscle contraction occurs following short stimulation periods.

To confirm that phosphorylation of TrkB.FL is ligand-specific, we analyzed the effect of 47/TrkB, an anti-TrkB antibody that extracellularly competes with the binding of endogenous TrkB to any ligand. We found that this inhibitor under presynaptic stimulation, with or without muscle contraction significantly decreases pTrkB.FL after 30 min (−35.61% ± 7.43, *p* < 0.05; −29.24% ± 3.51, *p* < 0.05; respectively, Figure 3D), indicating that pTrkB.FL levels are ligand-dependent. Likewise, the increase in TrkB.FL phosphorylation that occurs after 1 min of presynaptic stimulation without muscle contraction was also ligand-dependent, as 47/TrkB reduced significantly pTrkB.FL levels (−32.67% ± 4.84, *p* < 0.05). However, 47/TrkB did not change pTrkB.FL levels after 1 min of presynaptic stimulation with muscle contraction (−1.98% ± 2.92 *p* > 0.05). Possibly, phosphorylation of TrkB.FL in this condition is too low for 47/TrkB to decrease it further.

Similarly, we also determined the level of pTrkB.FL when muscles are incubated in exogenous BDNF at 30 min of stimulation. Unexpectedly, exogenous BDNF did not have any effect on pTrkB level in any condition (presynaptic stimulation without muscle contraction: 4.70 ± 4.38 *p* > 0.05; presynaptic stimulation with muscle contraction: 0.99 ± 2.53 *p* > 0.05; Figure 3E). 47/TrkB, which blocks endogenous BDNF binding, is able to decrease pTrkB.FL but exogenous BDNF does not increase it. This suggests that TrkB phosphorylation is only induced by endogenous BDNF. However, we cannot discount the possibility that exogenous BDNF could induce an effect through the ratio FL/T1 without the need to induce phosphorylation.

Altogether, these results show that activity modulates BDNF/TrkB signaling in a time-dependent manner. At short times (1–10 min), synaptic activity and muscle contraction regulate phosphorylation of TrkB.FL and, at longer times (30 min), the regulation involves altering the level of TrkB.T1 without effects on TrkB.FL phosphorylation. Within 10 min, presynaptic stimulation induces phosphorylation of TrkB.FL whereas muscle contraction decreases it by the action of phosphatases. These results suggest that muscle contraction performs a regulatory control on the TrkB.FL signaling. After 30 min of stimulation, neither synaptic activity nor muscle contraction has any effect over TrkB.FL phosphorylation. However, this prolonged postsynaptic activity regulates BDNF/TrkB signaling via a reduction of TrkB.T1. Next, we focused on the effects of decreased TrkB.T1 after 30 min of stimulation on the PKCs.

#### Muscle Contraction Promotes Changes in cPKC Isoforms α and βI through TrkB Receptor

**Figure 4:**
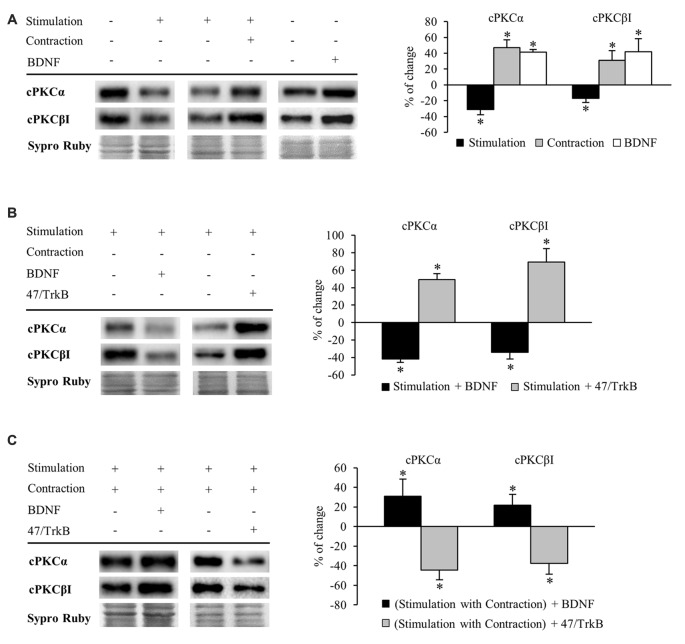
**Synaptic activity and muscle contraction modulates cPKC isoforms through BDNF/TrkB signaling**. Panels **(A–C)** show Western blot bands and their quantification. **(A)** cPKC isoforms α and βI in presynaptic stimulation treatment and contraction treatment at 1 Hz stimulation for 30 min. cPKC α and βI in basal conditions with exogenous BDNF for 30 min. Presynaptic stimulation has been simplified as Stimulation. Each column has been compared to its respective control (see Table 1). Results show that presynaptic stimulation decreased the levels of cPKC α and βI whereas muscle contraction increased them. In basal conditions, exogenous BDNF increased the levels of cPKC α and βI. **(B)** Effect of exogenous BDNF (10 μM) and 47/TrkB (10 μg/ml) on cPKC isoforms α and βI in presynaptic stimulation treatment. BDNF enhanced the effects of presynaptic stimulation, decreasing cPKC levels, and conversely, 47/TrkB increased them. **(C)** Effects of exogenous BDNF (10 μM) and 47/TrkB (10 μg/ml) on cPKCα and cPKCβI in presynaptic stimulation with contraction treatment. BDNF enhanced the effects of presynaptic stimulation with contraction treatment, increasing cPKC levels, and conversely, 47/TrkB decreased them. Data are mean percentage ± SEM, *p < 0.05. Abbreviations: cPKCα, conventional Protein Kinase C (PKC) α; cPKCβI, conventional PKC βI.

Once we had evaluated how BDNF and its receptors are modulated by activity, we proceeded to examine how this regulation extends to the two presynaptic cPKC isoforms (cPKCα and cPKCβI). TrkB tyr816 phosphorylation directly activates PLCγ1 (Middlemas et al., 1994) which, in turn, activates cPKC through DAG and Ca^2+^. Moreover, Besalduch et al. (2010) demonstrated that these isoforms are modulated by activity and that muscle contraction has a key role in their upregulation. Our results indicate that presynaptic stimulation resulted in a statistically significant decrease of cPKCα and cPKCβI protein levels (Figure 4A, newly reproduced data from Besalduch et al., 2010). This reduction in PKC levels could be due to its activation, and its subsequently degradation (Lee et al., 1996; Lu et al., 1998; Kang et al., 2000; Gould and Newton, 2008; Gould et al., 2009). Thus, we tested whether calcium-dependent PKC is affected by high (5 mM) extracellular Ca^2+^ when combined with presynaptic stimulation. As expected, high Ca^2+^ significantly decreased the level of cPKCα protein (−52.36 ± 2.02, *p* < 0.05) and cPKCβI (−29.15 ± 3.86, *p* < 0.05). Furthermore, we previously demonstrated that MARCKS phosphorylation (PKC’s substrate) is increased after presynaptic stimulation (Obis et al., 2015b). These results reinforce the fact that the reduction in cPKCα and cPKCβI promoted by synaptic activity could be due to PKC activation.

Conversely, muscle contraction significantly increased both cPKC isoforms, possibly to provide a pool ready to be activated (Figure 4A). These results suggest that a retrograde factor from the muscle cell could influence these isoforms in the nerve terminal. Thus, we proceeded to determine if this modulation is related to BDNF/TrkB signaling. We found that under basal conditions of no stimulation, incubation of exogenous BDNF significantly enhanced cPKCα and cPKCβI protein levels (Figure 4A), mimicking the effect of muscle contraction.

Exogenous BDNF decreased cPKCα and cPKCβI protein levels (−41.96% ± 3.66 *p* < 0.05; −34.21% ± 7.77 *p* < 0.05; respectively) under presynaptic stimulation (Figure 4B). This result may indicate that TrkB signaling reduces total cPKC levels in this condition, possibly due to an increase in their activation and subsequent degradation. In concordance, 47/TrkB preincubation reversed the effects of presynaptic stimulation, increasing cPKCα and cPKCβI protein levels a 49.10% ± 6.90 and a 69.10% ± 15.6, respectively (*p* < 0.05; Figure 4B). This increase could be explained because 47/TrkB inhibits TrkB signaling and decreases cPKC activation and their subsequent degradation.

On the other hand, under presynaptic stimulation with muscle contraction, exogenous BDNF further enhanced the increase of cPKCα and cPKCβI protein levels (30.83% ± 17.60, *p* < 0.05; 21.78% ± 11.09, *p* < 0.05, respectively; Figure 4C). In concordance, incubation with 47/TrkB completely reversed the effects produced by presynaptic stimulation with contraction, decreasing cPKCα and cPKCβI protein levels (−44.40% ± 10.0, *p* < 0.05; −37.60% ± 11.10, *p* < 0.05, respectively; Figure 4C). Thus, these results indicate that muscle contraction can enhance the levels of cPKCα and cPKCβI through BDNF/TrkB signaling, reverting the synaptic-induced downregulation of cPKC isoforms. Interestingly, at this point, muscle contraction significantly decreases TrkB.T1 levels without changing TrkB.FL. Therefore, the balance between TrkB.FL/TrkB.T1 could enhance cPKC synthesis or, alternatively, could inhibit its activity-induced degradation, increasing the total levels to revert the effect of synaptic activity.

Altogether, these results demonstrate a direct link between activity, BDNF/TrkB signaling and cPKCα and cPKCβI protein levels. In brief, BDNF regulation is directed by both synaptic activity and muscle contraction in opposite directions, emphasizing a key role of nerve-induced muscle contraction in the modulation of presynaptic cPKCα and cPKCβI isoforms through TrkB.

#### Phosphorylation of cPKC Isoforms (cPKCα and cPKCβI) is Regulated by Neuro-muscular Activity and TrkB

**Figure 5:**
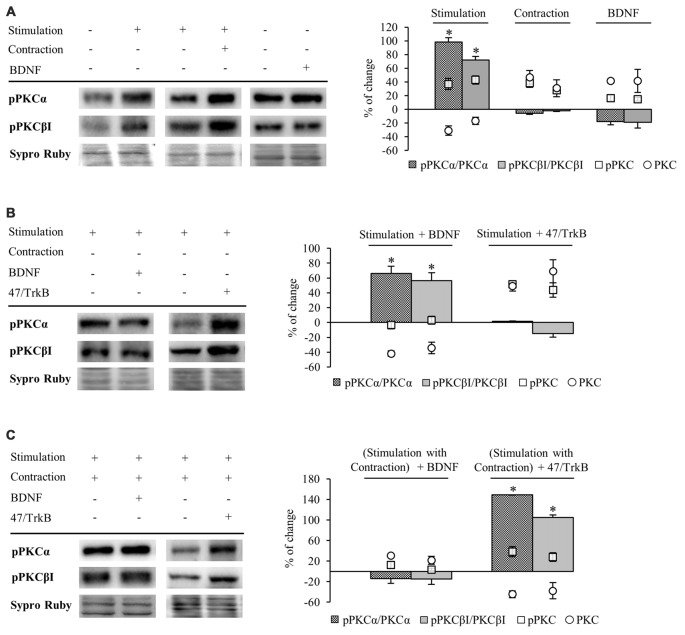
**Phosphorylation of cPKC isoforms is regulated by activity and TrkB**. Panels **(A–C)** show Western blot bands and their quantification. **(A)** pPKCα and pPKCβI in presynaptic stimulation treatment and contraction treatment at 1 Hz stimulation for 30 min. pPKCα and pPKCβI in basal conditions with exogenous BDNF for 30 min. Presynaptic stimulation has been simplified as Stimulation. Each column has been compared to its respective control (see Table 1). Results show that presynaptic stimulation increases the ratio of phosphorylated forms of cPKCα and cPKCβI. However, muscle contraction does not change the ratio of pPKC/PKC but increases the individual phosphorylated cPKCα and cPKCβI. Furthermore, the ratio of phosphorylated forms of cPKCα and cPKCβI did not show any change under basal conditions of no stimulation with exogenous BDNF. **(B)** Effect of exogenous BDNF (10 μM) and 47/TrkB (10 μg/ml) on pPKC isoforms α and βI in presynaptic stimulation treatment. Incubation with exogenous BDNF led to a significant increase of the ratio of pPKCα and pPKCβI (due to a decrease in the total amount of total cPKCα and cPKCβI). Incubation with 47/TrkB does not affect the ratio because the increase in the individual phosphorylated PKC is a consequence of the increase in total PKC. **(C)** Effects of exogenous BDNF (10 μM) and 47/TrkB (10 μg/ml) on pPKCα and pPKCβI in presynaptic stimulation with contraction treatment. Incubation with exogenous BDNF slightly decreases pPKC/PKC for either cPKCα and cPKCβI (with an accompanied increase in the total amount of PKC and the maintenance of pPKCα and pPKCβI). Incubation with 47/TrkB significantly increases the ratio of cPKCα and cPKCβI phosphorylation. In concrete, the decrease in total PKC is accompanied by an increase in the individual phosphorylation of PKC. Specific phosphorylation was determined as the ratio of phosphorylated protein to total protein content, but also as the individual protein phosphorylation level. Data are mean percentage ± SEM, *p < 0.05. Abbreviations: cPKCα, conventional- PKC α; cPKCβI, conventional- PKC βI; pPKCα, phosphorylated PKC α; pPKCβI, phosphorylated PKC βI.

Activation of PKC isoforms requires phosphorylation (Newton, 2003). Therefore, our next objective was to determine whether neuromuscular activity affects phosphorylation of cPKCα and cPKCβI isoforms. Measurements of a protein’s phosphorylation status may change through modification of the individual proteins phosphorylation level (Tremblay et al., 2007) and/or by alterations within the total amount of protein available (Yung et al., 2011). As such, we show specific phosphorylation determined as the ratio of phosphorylated protein to total amount of protein available, but also as the individual proteins phosphorylation level (Figure 5).

Presynaptic stimulation without muscle contraction resulted in a statistically significant increase of the ratio of phosphorylated forms of cPKCα (98.47% ± 4.33, *p* < 0.05) and cPKCβI (72.26% ± 2.07, *p* < 0.05; Figure 5A). This increase in pPKC levels indicates that presynaptic stimulation enhances directly phosphorylation of cPKC. Moreover, this increase in pPKC levels reaffirms the fact that the total PKC decrease described above is caused by an activation-induced degradation. Furthermore, muscle contraction does not change the ratio of pPKC/PKC either cPKCα or cPKCβI (Figure 5A). However, the individual phosphorylated cPKCα and cPKCβI levels are increased after muscle contraction (pPKCα 36.95% ± 8.13, *p* < 0.05; pPKCβI 42.98% ± 6.17, *p* < 0.05; Figure 5A) similarly than cPKCα and cPKCβI (Figure 4). In this condition, where total cPKC are increased, muscle contraction would inhibit activity-dependent degradation or, alternatively, enhance cPKC synthesis, establishing a larger pool of cPKCs isoforms ready to be phosphorylated and activated. This may indicate that muscle contraction enhances phosphorylation of cPKCs by means of increase of total protein PKC protein level. Moreover, we found that under basal conditions of no stimulation with exogenous BDNF, pPKCα and pPKCβI protein levels remain the same (Figure 5A).

We next determined the involvement of BDNF/TrkB pathway on pPKC levels. Preincubation with exogenous BDNF under presynaptic stimulation increases the ratio of pPKCα (66.32% ± 9.53, *p* < 0.05) and pPKCβI protein levels (56.50% ± 10.47, *p* < 0.05; Figure 5B). However, the increase in the ratio is due to a decrease in the total amount of total cPKCα and cPKCβI, indicating that the phosphorylated pool is maintained and exogenous BDNF does not promote PKC phosphorylation. Moreover, 47/TrkB does not affect the ratio because the increase in the individual phosphorylated PKC is a consequence of the increase in total PKC (Figure 5B). On the other hand, under presynaptic stimulation with muscle contraction, exogenous BDNF slightly decreases pPKC/PKC either cPKCα and cPKCβI. These decreases are due to an increase in the total amount of PKC with an accompanied maintenance of pPKCα and pPKCβI (Figure 5C). Thus, confirming that the contraction thought BDNF increase PKC synthesis rather than PKCs phosphorylation. However, incubation with 47/TrkB significantly increases the ratio of cPKCα and cPKCβI phosphorylation (149.01% ± 10.11, *p* < 0.05; 105.22% ± 8.86, *p* < 0.05, respectively; Figure 5C). In concrete, the decrease in total PKC is accompanied by an increase in the individual phosphorylation of PKC (38.32% ± 6.05, *p* < 0.05) and PKCβI (28 ± 4.92, *p* < 0.05). This might indicate that the activity-dependent ratio FL/T1 could decrease pPKCs due to an activity-dependent pPKC translocation, action and subsequent degradation.

So, an increase of the synaptic activity results in an increase of the phosphorylation of cPKCα and cPKCβI isoforms, whose activity would be enhanced by TrkB/PLCγ signaling pathway, decreasing total cPKCα and cPKCβI levels due to their subsequent degradation. Muscle contraction, through an increase in TrkB FL/T1 ratio would inhibit PKC degradation or enhance their synthesis, increasing the total PKC levels ready to be phosphorylated to revert the downregulating effect of the synaptic activity.

#### cPKCβI Is Involved in ACh Release in the NMJ

**Figure 6:**
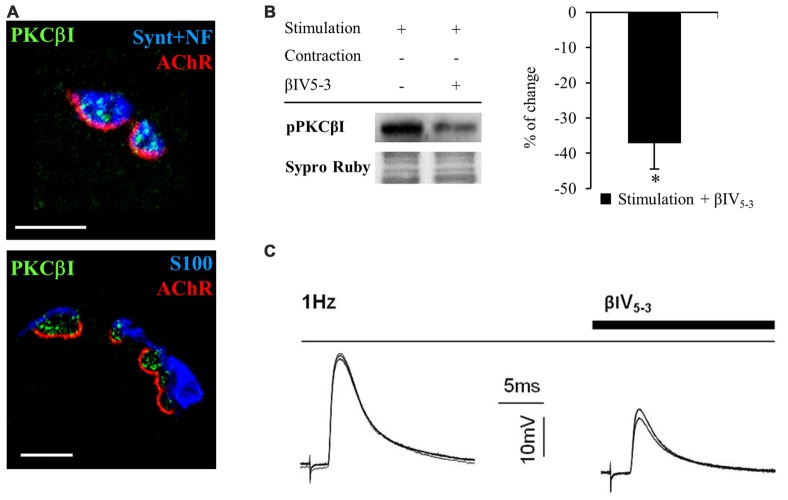
**cPKCBI is involved in ACh release in the neuromuscular junction (NMJ). (A)** Semithin cross-sections from whole-mount multiple-immunofluorescent stained levator auris longus (LAL) muscles. (**A**, top) Triple staining labeled cPKCβI (in green), AChRs (fluorescent α-BTX in red) and nerve terminal (with anti-syntaxin and anti-neurofilament antibodies in blue). (**A**, bottom) Triple staining labeled cPKCβI in green, AChRs in red and Schwann cell (with an anti S100 antibody in blue). The images show the cPKCβI immunolabel located only between Schwann cell and muscle cell and well colocalized with the nerve terminal, indicating that cPKCβI is exclusively located at the presynaptic component. The scale bars indicate 2.5 μm. **(B)** Western blot bands and their quantification show the effect of the selective translocation peptide inhibitor (βIV5–3, 10 μM) on pPKCβI in the membrane fraction in presynaptic stimulation treatment. Incubation with βIV5–3 decreased the phosphorylated cPKCβI in the membrane fraction, thus confirming its inhibitory effect. Data are mean percentage ± SEM, *p < 0.05. **(C)** Intracellular recordings show evoked Endplate potentials (EPPs) in basal conditions (1 Hz) and after incubation with βIV5–3. Raw data indicate that ACh release is reduced by incubation with βIV5–3, highlighting the involvement of cPKCβI in neurotransmitter release. Abbreviations: LAL, levator auris longus; α-BTX, α-bungarotoxin; cPKCβI, conventional-PKC βI; pPKCβI, phosphorylated PKC βI; EPP, end-plate potential.

PKC is an important family of kinases that regulates neurotransmission at the NMJ (Hori et al., 1999; Santafé et al., 2005, 2006). In particular, PKC is activated and regulates the release of ACh when electrical stimulation is applied to the nerve (Besalduch et al., 2010). However, it is unknown which isoform of PKC is involved in this regulation. cPKCα and cPKCβI are differently distributed in the NMJ with cPKCα located in the nerve terminal, muscle cell and terminal Schwann cells but cPKCβI is exclusively located in the nerve terminal (Besalduch et al., 2010). When we examine the expression of cPKCβI based on immunofluorescent labeling of semithin cross-sections of LAL muscles (Figure 6A), we find cPKCβI fine granular green immunofluorescence located over the postsynaptic line of the nicotinic acetylcholine receptor (nAChR) site (in red) and colocalized with the neurofilament and syntaxin (NF+Synt, in blue top image 1) in the nerve terminal. cPKCβI is not colocalized with labels for either Schwann cells (S100, in blue bottom image 2) or AChRs postsynaptically (in red). These results confirm the exclusive presynaptic localization of cPKCβI and makes it a good candidate to regulate neurotransmitter release.

To test this idea directly, we used an isozyme-selective translocation peptide inhibitor (βIV5–3; Liu et al., 1999; Zhang et al., 2015) to inhibit cPKCβI activity. Western blotting confirmed that βIV5–3 decreases the ratio of phosphorylated cPKCβI levels (−36.95% ± 7.60, *p* < 0.05; Figure 6B) in the membrane fraction of rat diaphragm muscles. Electrophysiological experiments in presynaptic stimulated muscles (contraction blocked) incubated with βIV5–3 in the dose range commonly used (1, 5, and 10 μM, 1 h incubation) revealed a significant reduction of EPP amplitude, indicating significantly less ACh release (Figure 6C). Altogether, these results highlight the key role of cPKCβI in neurotransmission in NMJ.

### Discussion

In the neuromuscular system, evidence supports BDNF/TrkB signaling as a regulator of neuromuscular function. However, it remained unknown if nerve-induced muscle contraction *per se* can modulate crucial aspects of neuromuscular synaptic function through BDNF and its receptor, TrkB. The results of the present study demonstrate that nerve induced-muscle activity is a key regulator of BDNF/TrkB signaling pathway, activating presynaptic cPKC isoforms (in particular cPKCβI) to modulate synaptic function.

Diaphragm muscle has been described as a useful model of flat skeletal muscle to study synaptic function (Rosato Siri and Uchitel, 1999; Urbano et al., 2003; Besalduch et al., 2010; Chand et al., 2015; Obis et al., 2015b). Accessibility to the phrenic nerve helps to dissect and stimulate it and thus enhance independently synaptic activity and muscle contraction. Diaphragm is in a way unusual because only a subset of muscle fibers is active with a prolonged duty cycle as compared to other skeletal muscles (Mantilla et al., 2004, 2014; Seven et al., 2014). However, nerve stimulation simultaneously recruits all motor units and uniforms the heterogeneous level of activity of the fibers.

#### Nerve-Induced Muscle Contraction Enhances the Activity-Dependent Increase of mBDNF and Downregulates TrkB.T1 Levels in Skeletal Muscle

**Figure 7:**
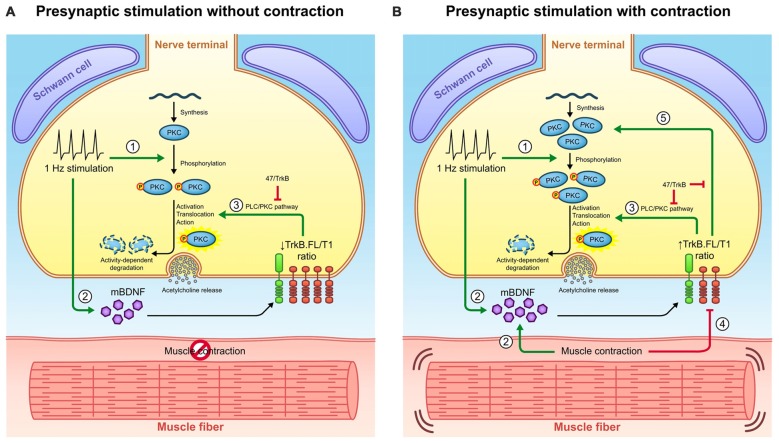
**Retrograde communication from myocytes to axon terminals through BDNF/TrkB signaling. (A)** Graphical representation of BDNF signaling outcomes observed under presynaptic stimulation without muscle contraction and presynaptic stimulation with muscle contraction. Presynaptic activity (1) promotes cPKC phosphorylation and (2) increases BDNF levels. BDNF acts presynaptically through (3) TrkB/PLCγ/PKC pathway to complete the activation of pPKC and thus enhance synaptic vesicle fusion and ACh release. Once PKC have executed their action, they are typically degraded thus decreasing its protein levels. **(B)** Graphical representation of BDNF signaling outcomes observed under presynaptic stimulation with contraction. The presence of contraction (2) further increases BDNF protein levels and (4) decreases TrkB.T1 protein levels, ultimately increasing the ratio TrkB.FL/T1. Even though this ratio still enhances (3) cPKC activity and its subsequent degradation, it promotes the increase in total PKC protein levels (5). Presumably, this might be due to an increase in PKC synthesis or alternatively, to a decrease in their activity-induced degradation. Consequently, pPKC levels are enhanced. The influence of TrkB.FL/T1 ratio over cPKC in both conditions is demonstrated by the sequestering antibody 47/TrkB. Therefore, muscle contraction could modulate BDNF/TrkB signaling to promote a retrograde regulatory feedback from the myocyte that maintains the levels of presynaptic cPKC. This may have an impact in NMJ functionality since cPKCβI activity is required for acetycholine release.

Exercise training increases BDNF expression in spinal cord and in skeletal muscle in rodents (Gómez-Pinilla et al., 2001, 2002; Cuppini et al., 2007; Zoladz and Pilc, 2010; Gomez-Pinilla et al., 2012) and basal levels of neuromuscular activity are required to maintain normal levels of BDNF in the neuromuscular system (Gómez-Pinilla et al., 2002). However, previous studies did not explore if BDNF expression in muscle was enhanced by synaptic activity independent of muscle contraction or whether muscle contraction was also necessary. Here we show that both activities can increase muscle BDNF levels, with muscle contraction being able to increase levels over and above what nerve transmission alone can enhance. Several findings suggest the muscle cell as a source of BDNF. Although BDNF is found in the three cells at the NMJ (Garcia et al., 2010), *bdnf* mRNA is only located inside myocytes (Liem et al., 2001), and not in the presynaptic region of the axon. Moreover, BDNF is produced by contracting myotubes *in vitro* (Matthews et al., 2009). By using μ-CgTx-GIIIB we are able to block myofibril contraction without altering ACh signaling. We show here that the increase of BDNF levels induced by presynaptic stimulation could arise from the presynaptic region (due to the electrical stimulus) and/or in the postsynaptic region (due to local ACh signaling). However, the greater increase of BDNF levels when both nerve and muscle are active compared to when only the synapse is active strongly suggests a postsynaptic origin through *bdnf* mRNA translation that is directly linked to myofibril contraction (represented as via 2 in Figure 7). Moreover, evidence indicates that neurotrophins are released acutely following neuronal depolarization (Griesbeck et al., 1999; Mowla et al., 1999; Goggi et al., 2003). In fact, direct activity-dependent pre- to post-synaptic transneuronal transfer of BDNF has recently been demonstrated using fluorescently-labeled BDNF (Kohara et al., 2001). Moreover, electrical stimulation can activate Schwann cells (SCs) to secrete BDNF, which requires the involvement of calcium influx (Luo et al., 2014). Because the well-known interactions between Schwann cell and the nerve terminal (Todd et al., 2007), some involvement of the glial cell in the responses we observed would be not discarded. Thus, although the source and the realize of BDNF is not clear, our results show that activity in either the synapse or the muscle increase mature BDNF in skeletal muscle.

Previous results indicate that *bdnf* mRNA increases in skeletal muscle after several days of increased physical activity (Gómez-Pinilla et al., 2001, 2002; Cuppini et al., 2007; Zoladz and Pilc, 2010; Gomez-Pinilla et al., 2012). However, we find that 30 min of synaptic activity with or without contraction was not sufficient to increase *bdnf* mRNA in muscle, suggesting that short-term acute neuromuscular activity enhances muscle BDNF levels by promoting its translation and/or maturation (e.g., through increased protease activity), allowing a build-up of mBDNF and leaving net pro-BDNF levels unchanged.

BDNF isoforms, pro-BDNF and mBDNF, bind distinct receptors to mediate divergent neuronal actions (Lu, 2003; Woo et al., 2005; Hempstead, 2006; Yang et al., 2009; Je et al., 2013). Pro-BDNF interacts preferentially with p75, whereas mBDNF selectively binds and activates its specific receptor TrkB. There are several alternatively spliced isoforms of TrkB with the same affinity to neurotrophins, including TrkB.FL and two truncated TrkB isoforms T1 and T2 (TrkB.T1 and TrkB.T2), which lack part of the intracellular kinase domain (Middlemas et al., 1991; Reichardt, 2006). Evidence suggests that heterodimers of TrkB.FL with the truncated isoforms inhibit trans-autophosphorylation of TrkB.FL, reducing BDNF signaling (Eide et al., 1996; Baxter et al., 1997; Rose et al., 2003; Dorsey et al., 2012; Wong and Garner, 2012). TrkB.T2 is a variant mainly predominant in the brain tissue and does not appear to have individual signaling ability (Stoilov et al., 2002). TrkB.T1 is the main truncated isoform in the skeletal muscle and some studies suggest unique signaling roles for TrkB.T1, for example, by modulating Ca^2+^ signaling mechanisms (Rose et al., 2003). Other evidence suggests that TrkB.T1 acts in a dominant negative fashion to decrease signaling through TrkB.FL (Eide et al., 1996; Gonzalez et al., 1999; Haapasalo et al., 2001; Dorsey et al., 2012). In concordance with previous findings (Dorsey et al., 2012), we show that TrkB.T1 levels predominate over TrkB.FL isoform 11:1 in the resting NMJ (Figure 2A). We also found that muscle contraction downregulates TrkB.T1 (decreasing the ratio to 6:1) without changing TrkB.FL or p75 levels (represented as via 4 in Figure 7). Therefore, it appears that the ratio between TrkB.FL and TrkB.T1 could determine the net effect of BDNF signaling at neuromuscular system.

BDNF binding to the TrkB.FL activates the intrinsic tyrosine kinase domain, leading to autophosphorylation in the activation loop (tyr701, tyr706 and tyr707; Guiton et al., 1994; Reichardt, 2006). The phosphorylation of these residues can leads to the transphosphorylation of others tyrosine residues (Cunningham et al., 1997; Friedman and Greene, 1999) being tyr515 and tyr816 the most extensively studied phosphorylation sites (Middlemas et al., 1994; Segal et al., 1996). pTyr515 interacts with Shc or Frs2 and provides a mechanism for the activation of the: (i) Ras–mitogen-activated protein kinase (Ras/MAPK) pathway; and (ii) phosphatidylinositol-3 kinase—Akt pathway (PI-3K/Akt). On the other hand, pTyr816 links TrkB receptors to the (iii) phospholipase Cγ gamma (PLC-γ) pathway (Obermeier et al., 1993). Although the other signal transduction pathways activated by TrkB could be involved in BDNF response (e.g., due to phosphorylation of tyr515), activation of PLCγ is one attractive candidate to mediate synaptic potentiation by PKC because its activation would result in intracellular Ca^2+^ release via the second messenger IP_3_ (Obermeier et al., 1993). Moreover, it is known that, in most cells, M_1,_ A_1_, (autoreceptors of the principal transmitter and cotransmitter product adenosine) and TrkB receptors among others, cooperate by stimulating PLCγ pathway. In this regard, it should also take into account the possible involvement of M_1_, A_1_ in PKC activation in the NMJ.

Neuronal activity has been shown to rapidly activate TrkB and potentiate its signaling, an effect attributed to activity-dependent secretion of BDNF (Meyer-Franke et al., 1998; Aloyz et al., 1999; Patterson et al., 2001). However, Du et al. (2003) reported that the activity-dependent enhancement of TrkB tyrosine kinase in cultured hippocampal neurons is not due to elevated BDNF secretion. Moreover, it has been demonstrated that the responsiveness of TrkB phophorylation to BDNF is reduced after the 2nd week postnatally in rat brain microslices at sites tyr816, tyr515 and tyr705/6 (Di Lieto et al., 2012). In our experiments, exogenous BDNF did not increase the tyr816 phosphorylation of TrkB.FL, known to trigger the PLCγ signaling pathway that activates PKC. Nevertheless, we found that presynaptic stimulation induces a quick increase in pTrkB.FL after 1–10 min but returned to baseline by 30 min (Figure 3B). On the one hand, the rapid phosphorylation of TrkB (1 min) that we found, suggests that the action of BDNF is particularly fast. This could be due to a quick extracellular diffusion of BDNF or to a site of release close to the NMJ. Both possibilities could explain the short time course of presynaptic TrkB activation. On the other hand, the subsequent decrease in the phosphorylation of TrkB could be caused by a decrease in the phosphorylation process or by phosphatase activity. We found support for enhanced phosphatase activity: muscle contraction at short times decreases phosphorylation of TrkB.FL by increasing phosphatase activity (Figures 3B,C). Previous work revealed the complexity of phosphatase control and showed that endogenous protein-tyrosine phosphatases negatively control BDNF sensitivity, antagonizing tyrosine phosphorylation of TrkB (Rusanescu et al., 2005; Ambjørn et al., 2013; Gatto et al., 2013; Ozek et al., 2014). The mechanism by which muscle contraction decreases TrkB phosphorylation (tyr 816) at short times at the NMJ needs further investigation. This mechanism could exert a retrograde regulatory control over the BDNF/TrkB.FL signaling to modulate presynaptic BDNF/TrkB action. In conclusion, the modulation of BDNF/TrkB signaling by neuromuscular activity is time-dependent at the NMJ. At short times (1–10 min), synaptic activity and muscle contraction regulate phosphorylation of TrkB.FL (tyr816) and, at longer times (30 min), the regulation is mediated by an effect on TrkB.T1.

#### Muscle Contraction Induced by Nerve Electrical Activity Promotes Changes in cPKC Isoforms through BDNF/TrkB Pathway

Several studies demonstrated that BDNF-induced potentiation of presynaptic vesicle release requires TrkB phosphorylation and PLC activation (Kleiman et al., 2000), which activates PKC. When PKCs are activated (phosphorylated and anchored to the membrane) they enhance vesicle fusion and ACh release (West et al., 1991; Numann et al., 1994; Byrne and Kandel, 1996; Besalduch et al., 2010; Lanuza et al., 2014).

Recently, we reported that synaptic activity induces changes in the presynaptic expression of the novel PKCε through TrkB receptor at the NMJ (Obis et al., 2015a). As previously published (Besalduch et al., 2010), here we also show that cPKCα and cPKCβI are downregulated by presynaptic stimulation (represented as via 3 in Figure 7). In this presynaptic stimulation condition, MARCKS phosphorylation levels (PKC’s substrate) are increased, an indicator of PKC activation (Obis et al., 2015b). Once PKCs are activated and have executed their action, PKCs are down-regulated in an activation-dependent manner, a process mediated by the proteasome (Lee et al., 1996; Lu et al., 1998; Kang et al., 2000; Gould and Newton, 2008; Gould et al., 2009). As we show here, high Ca^2+^ conditions induce a decrease in calcium dependent cPKCα and cPKCβI protein levels, indicating that they are activated and then degraded without being replaced by newly synthesized cPKCs.

In contrast, muscle contraction increases cPKCα and cPKCβI protein levels through TrkB and reverses the downregulation induced by the synaptic activity. This could be associated with an increased protein synthesis (represented as via 5 in Figure 7) or decreased protein degradation (represented as via 3 in Figure 7). As far as we know, the mechanism of the synthesis of cPKC has not been extensively studied. However, it is known that BDNF has effects on the proteome and it may be due to changes in transcription activity (e.g., activating CREB; Finkbeiner et al., 1997; Groth and Mermelstein, 2003; Caldeira et al., 2007). Moreover, it may be due to a direct regulation of the translation machinery through the mammalian target of rapamycin (mTOR) pathway (Takei et al., 2001). A plausible hypothesis could be that some of these signaling pathways could be also involved in the BDNF induced-enhancement of presynaptic cPKC at the NMJ.

cPKCα and cPKCβI are presynaptically located, with cPKCβI being exclusively located at the nerve terminal (Besalduch et al., 2010). Hence, the modulation of presynaptic cPKCα and cPKCβI may require a neurotrophic positive feedback generated by the postsynaptic contractile activity, BDNF being a possible mediator. It has been suggested that neuromuscular activity increases retrograde transport of BDNF from the muscle to the spinal cord (Yan et al., 1992; Koliatsos et al., 1993; Curtis et al., 1998; Sagot et al., 1998).

Moreover, our results demonstrate that synaptic activity and muscle activity have opposite effects on cPKC protein level and these effects are mediated by the increased endogenous BDNF (induced by pre- and postsynaptic activities) through TrkB. This suggests distinct roles of presynaptic vs. postsynaptic induced-BDNF. Moreover, exogenous BDNF enhances the opposite effects of presynaptic and postsynaptic activities on cPKC protein levels through a different ratio of TrkB.FL/T1. Muscle contraction increases TrkB.FL/T1 ratio and the reduction of the T1 dominant negative fashion over FL signaling upregulates presynaptic cPKCs. Therefore, the apparent distinct roles of the pre- and postsynaptic BDNF are consequence of the muscle contraction-induced decrease of TrkB.T1. This TrkB.T1 downregulation could be related with the recycling events of the receptor. TrkB isoforms seem to be differentially recycled after BDNF-induced endocytosis with TrkB.FL receptor degraded (targeted to the lysosomes) more quickly than TrkB.T1 (Huang et al., 2009). Moreover, TrkB.T1 regulates extracellular BDNF levels in the brain and binds, internalizes and presents BDNF to neurons via a spatial- and temporal-dependent mechanism (Biffo et al., 1995; Fryer et al., 1997; Alderson et al., 2000). Some event related with TrkB.T1 mediated sequestration and recycling of BDNF may be involved in the distinct roles of presynaptic vs. postsynaptic BDNF.

#### cPKCβI is Involved in ACh Release at the NMJ

PKC isoforms need to be phosphorylated to be active (Newton, 2003). It is well documented that PKCs play an important role in the regulation of transmitter release (West et al., 1991; Numann et al., 1994; Byrne and Kandel, 1996; Catterall, 1999; Santafé et al., 2005, 2006). Our results show that presynaptic stimulation directly increases cPKCα and cPKCβ phosphorylation (represented as via 1 in Figure 7), to regulate neurotransmission release. However, after muscle contraction cPKCα and cPKCβ phosphorylation is may further increased due to the increase of their synthesis (represented as via 5 in Figure 7). There is also functional evidence indicating that TrkB regulates ACh release via the PKC pathway (Santafé et al., 2014). Our data show that cPKCβI isoform is decisively involved in regulating ACh release induced by electrical stimulation.

Together, the data support a regulatory mechanism which has been represented in Figure 7. Presynaptic activity (1) directly promotes cPKC phosphorylation and (2) increases BDNF levels. BDNF acts presynaptically through (3) TrkB/PLCγ/PKC pathway to complete the activation of pPKC and thus enhance synaptic vesicle fusion and ACh release. Once PKC have executed their action, they are typically degraded thus decreasing its protein levels. The presence of contraction (2) further increases BDNF protein levels and (4) decreases TrkB.T1 protein levels, ultimately increasing the ratio TrkB.FL/T1. Even though this ratio still enhances (3) cPKC activity and its subsequent degradation, it promotes the increase in total PKC protein levels (5). Presumably, this might be due to an increase in PKC synthesis or alternatively, to a decrease in their activity-induced degradation. Thus, pPKC levels are enhanced. Consequently, presynaptic PKCs are enhanced in the nerve terminal and this establishes a larger pool of cPKC isoforms ready to promote neuromuscular transmission. This may have an impact in NMJ functionality since cPKCβI activity is required for acetylcholine release.

Several neuromuscular disorders show progressive loss of the connection between nerve and muscle. This leads to the pathological non-communication of the two tissues, eventually resulting in muscle weakness. Our results suggest that a decrease in neuromuscular activity, as it occurs in most neuromuscular disorders, could affect the BDNF/TrkB retrograde pathway linking pre- and postsynaptic components to correctly maintain neuromuscular function.

### Author Contributions

EH: data collection, quantitative analysis, literature search, data interpretation and statistics; VC: data collection, literature search, data interpretation and design graphic abstract; TO: electrophysiological analysis; LN, AS, MT and MMS: data collection; KH and CLJ: PCR experiments and data interpretation; JT, MAL and NG: conception and design, literature search, data interpretation and manuscript preparation.

## Conflict of Interest Statement

The authors declare that the research was conducted in the absence of any commercial or financial relationships that could be construed as a potential conflict of interest.

## Abbreviations

ACh: Acetylcholine
AChRs: acetylcholine receptors
BSA: bovine serum albumin
BDNF: brain-derived neurotrophic factor
CNS: central nervous system
cPKC: conventional protein kinase C
cPKC cPKCα: conventional protein kinase C alpha
cPKC βI: conventional protein kinase C beta I
βIV5–3: cPKCβI-specific translocation inhibitor peptide
EPPs: evoked endplate potentials
GAPDH: glyceraldehyde-3-phosphate dehydrogenase
HRP: horseradish peroxidase
LAL: levator auris longus
MAPK: mitogen-activated protein kinase
MARCKS: myristoylated alanine-rich C-kinase substrate
NMJ: neuromuscular junction
NT-4: neurotrophin-4
p75: p75 neurotrophin receptor
PI3K: phosphatidylinositol 3-kinase
PBS: phosphate buffer saline
PLC: phospholipase C
PVDF: polyvinylidene difluoride
qRT-PCR: quantitative reverse transcriptase polymerase chain reaction
RACK: receptor for activated C-kinase
MEPPs: spontaneous miniature endplate potentials
TSBT: tris-buffered saline, Tween 20
TrkB: tropomyosin receptor kinase B
TrkB.FL: tropomyosin receptor kinase B full length isoform
TrkB.T1: tropomyosin receptor kinase B truncated isoform 1
TrkB.T2: tropomyosin receptor kinase B truncated isoform 2
α-BTX: α-bungarotoxin
μ-CgTx-GIIIB: μ-conotoxin GIIIB.

## References

[B1] Alderson R. F. Curtis R. Alterman A. L. Lindsay R. M. DiStefano P. S. (2000). Truncated TrkB mediates the endocytosis and release of BDNF and neurotrophin-4/5 by rat astrocytes and schwann cells *in vitro*. Brain Res. 871, 210–222. 10.1016/s0006-8993(00)02428-8 10899288

[B2] Aldridge G. M. Podrebarac D. M. Greenough W. T. Weiler I. J. (2008). The use of total protein stains as loading controls: an alternative to high-abundance single-protein controls in semi-quantitative immunoblotting. J. Neurosci. Methods 172, 250–254. 10.1016/j.jneumeth.2008.05.003 18571732 PMC2567873

[B3] Aloyz R. Fawcett J. P. Kaplan D. R. Murphy R. A. Miller F. D. (1999). Activity-dependent activation of TrkB neurotrophin receptors in the adult CNS. Learn. Mem. 6, 216–231. 10492004 PMC311290

[B4] Ambjørn M. Dubreuil V. Miozzo F. Nigon F. Møller B. Issazadeh-Navikas S.. (2013). A loss-of-function screen for phosphatases that regulate neurite outgrowth identifies PTPN12 as a negative regulator of TrkB tyrosine phosphorylation. PLoS One 8:e65371. 10.1371/journal.pone.0065371 23785422 PMC3681791

[B5] Baldwin K. M. Haddad F. Pandorf C. E. Roy R. R. Edgerton V. R. (2013). Alterations in muscle mass and contractile phenotype in response to unloading models: role of transcriptional/pretranslational mechanisms. Front. Physiol. 4:284. 10.3389/fphys.2013.00284 24130531 PMC3795307

[B6] Barde Y. A. (1990). The nerve growth factor family. Prog. Growth Factor Res. 2, 237–248. 10.1016/0955-2235(90)90021-b 2133291

[B7] Baxter G. T. Radeke M. J. Kuo R. C. Makrides V. Hinkle B. Hoang R.. (1997). Signal transduction mediated by the truncated TrkB receptor isoforms, TrkB.T1 and TrkB.T2. J. Neurosci. 17, 2683–2690. 9092589 10.1523/JNEUROSCI.17-08-02683.1997 PMC6573096

[B8] Besalduch N. Tomàs M. Santafé M. M. Garcia N. Tomàs J. Lanuza M. A. (2010). Synaptic activity-related classical protein kinase C isoform localization in the adult rat neuromuscular synapse. J. Comp. Neurol. 518, 211–228. 10.1002/cne.22220 19937712

[B9] Bibel M. Barde Y. A. (2000). Neurotrophins: key regulators of cell fate and cell shape in the vertebrate nervous system. Genes Dev. 14, 2919–2937. 10.1101/gad.841400 11114882

[B10] Biffo S. Offenhäuser N. Carter B. D. Barde Y. A. (1995). Selective binding and internalisation by truncated receptors restrict the availability of BDNF during development. Development 121, 2461–2470. Available online at: http://dev.biologists.org/content/121/8/2461.long [Accessed April 16, 2017]. 7671810 10.1242/dev.121.8.2461

[B11] Byrne J. H. Kandel E. R. (1996). Presynaptic facilitation revisited: state and time dependance. J. Neurosci. 16, 425–435. 8551327 10.1523/JNEUROSCI.16-02-00425.1996 PMC6578640

[B12] Caldeira M. V. Melo C. V. Pereira D. B. Carvalho R. Correia S. S. Backos D. S.. (2007). Brain-derived neurotrophic factor regulates the expression and synaptic delivery of -amino-3-hydroxy-5-methyl-4-isoxazole propionic acid receptor subunits in hippocampal neurons. J. Biol. Chem. 282, 12619–12628. 10.1074/jbc.M700607200 17337442

[B13] Catterall W. A. (1999). Interactions of presynaptic Ca^2+^ channels and snare proteins in neurotransmitter release. Ann. N Y Acad. Sci. 868, 144–159. 10.1111/j.1749-6632.1999.tb11284.x 10414292

[B14] Chand K. K. Lee K. M. Schenning M. P. Lavidis N. A. Noakes P. G. (2015). Loss of β2-laminin alters calcium sensitivity and voltage-gated calcium channel maturation of neurotransmission at the neuromuscular junction. J. Physiol. 593, 245–265. 10.1113/jphysiol.2014.284133 25556799 PMC4293066

[B15] Cisterna B. A. Cardozo C. Sáez J. C. (2014). Neuronal involvement in muscular atrophy. Front. Cell. Neurosci. 8:405. 10.3389/fncel.2014.00405 25540609 PMC4261799

[B16] Cunningham M. E. Stephens R. M. Kaplan D. R. Greene L. A. (1997). Autophosphorylation of activation loop tyrosines regulates signaling by the TRK nerve growth factor receptor. J. Biol. Chem. 272, 10957–10967. 10.1074/jbc.272.16.10957 9099755

[B17] Cuppini R. Sartini S. Agostini D. Guescini M. Ambrogini P. Betti M.. (2007). Bdnf expression in rat skeletal muscle after acute or repeated exercise. Arch. Ital. Biol. 145, 99–110. 10.4449/aib.v145i2.868 17639782

[B18] Curtis R. Tonra J. R. Stark J. L. Adryan K. M. Park J. S. Cliffer K. D.. (1998). Neuronal injury increases retrograde axonal transport of the neurotrophins to spinal sensory neurons and motor neurons via multiple receptor mechanisms. Mol. Cell. Neurosci. 12, 105–118. 10.1006/mcne.1998.0704 9790733

[B19] Di Lieto A. Rantamäki T. Vesa L. Yanpallewar S. Antila H. Lindholm J.. (2012). The responsiveness of TrkB to BDNF and antidepressant drugs is differentially regulated during mouse development. PLoS One 7:e32869. 10.1371/journal.pone.0032869 22396798 PMC3292581

[B20] Dorsey S. G. Lovering R. M. Renn C. L. Leitch C. C. Liu X. Tallon L. J.. (2012). Genetic deletion of TrkB.T1 increases neuromuscular function. Am. J. Physiol. Cell Physiol. 302, C141–C153. 10.1152/ajpcell.00469.2010 21865582 PMC3328911

[B21] Du Y. Fischer T. Z. Lee L. N. Lercher L. D. Dreyfus C. F. (2003). Regionally specific effects of BDNF on oligodendrocytes. Dev. Neurosci. 25, 116–126. 10.1159/000072261 12966210

[B22] Eide F. F. Vining E. R. Eide B. L. Zang K. Wang X. Y. Reichardt L. F. (1996). Naturally occurring truncated TrkB receptors have dominant inhibitory effects on brain-derived neurotrophic factor signaling. J. Neurosci. 16, 3123–3129. 8627351 10.1523/JNEUROSCI.16-10-03123.1996 PMC2710135

[B23] Favreau P. Le Gall F. Benoit E. Molgó J. (1999). A review on conotoxins targeting ion channels and acetylcholine receptors of the vertebrate neuromuscular junction. Acta Physiol. Pharmacol. Ther. Latinoam. 49, 257–267. 10797869

[B24] Finkbeiner S. Tavazoie S. F. Maloratsky A. Jacobs K. M. Harris K. M. Greenberg M. E. (1997). CREB: a major mediator of neuronal neurotrophin responses. Neuron 19, 1031–1047. 10.1016/s0896-6273(00)80395-5 9390517

[B25] Friedman W. J. Greene L. A. (1999). Neurotrophin signaling via trks and p75. Exp. Cell Res. 253, 131–142. 10.1006/excr.1999.4705 10579918

[B26] Fryer R. H. Kaplan D. R. Kromer L. F. (1997). Truncated trkB receptors on nonneuronal cells inhibit BDNF-induced neurite outgrowth *in vitro*. Exp. Neurol. 148, 616–627. 10.1006/exnr.1997.6699 9417837

[B27] Garcia N. Tomàs M. Santafe M. M. Lanuza M. A. Besalduch N. Tomàs J. (2010). Localization of brain-derived neurotrophic factor, neurotrophin-4, tropomyosin-related kinase b receptor and p75 NTR receptor by high-resolution immunohistochemistry on the adult mouse neuromuscular junction. J. Peripher. Nerv. Syst. 15, 40–49. 10.1111/j.1529-8027.2010.00250.x 20433604

[B28] Gatto G. Dudanova I. Suetterlin P. Davies A. M. Drescher U. Bixby J. L.. (2013). Protein tyrosine phosphatase receptor type O inhibits trigeminal axon growth and branching by repressing trkB and ret signaling. J. Neurosci. 33, 5399–5410. 10.1523/JNEUROSCI.4707-12.2013 23516305 PMC3667612

[B29] Goggi J. Pullar I. A. Carney S. L. Bradford H. F. (2003). The control of [125I]BDNF release from striatal rat brain slices. Brain Res. 967, 201–209. 10.1016/s0006-8993(03)02225-x 12650981

[B30] Gomez-Pinilla F. Hillman C. (2013). The influence of exercise on cognitive abilities. Compr. Physiol. 3, 403–428. 10.1002/cphy.c110063 23720292 PMC3951958

[B31] Gomez-Pinilla F. Vaynman S. Ying Z. (2008). Brain-derived neurotrophic factor functions as a metabotrophin to mediate the effects of exercise on cognition. Eur. J. Neurosci. 28, 2278–2287. 10.1111/j.1460-9568.2008.06524.x 19046371 PMC2805663

[B32] Gómez-Pinilla F. Ying Z. Opazo P. Roy R. R. Edgerton V. R. (2001). Differential regulation by exercise of BDNF and NT-3 in rat spinal cord and skeletal muscle. Eur. J. Neurosci. 13, 1078–1084. 10.1046/j.0953-816x.2001.01484.x 11285004

[B33] Gómez-Pinilla F. Ying Z. Roy R. R. Molteni R. Edgerton V. R. (2002). Voluntary exercise induces a BDNF-mediated mechanism that promotes neuroplasticity. J. Neurophysiol. 88, 2187–2195. 10.1152/jn.00152.2002 12424260

[B34] Gomez-Pinilla F. Ying Z. Zhuang Y. (2012). Brain and spinal cord interaction: Protective effects of exercise prior to spinal cord injury. PLoS One 7:e32298. 10.1371/journal.pone.0032298 22384207 PMC3284558

[B35] Gonzalez M. Ruggiero F. P. Chang Q. Shi Y. J. Rich M. M. Kraner S.. (1999). Disruption of Trkb-mediated signaling induces disassembly of postsynaptic receptor clusters at neuromuscular junctions. Neuron 24, 567–583. 10.1016/s0896-6273(00)81113-7 10595510

[B36] Gould C. M. Kannan N. Taylor S. S. Newton A. C. (2009). The chaperones Hsp90 and Cdc37 mediate the maturation and stabilization of protein kinase C through a conserved PXXP motif in the C-terminal tail. J. Biol. Chem. 284, 4921–4935. 10.1074/jbc.m808436200 19091746 PMC2643500

[B37] Gould C. M. Newton A. C. (2008). The life and death of protein kinase C. Curr. Drug Targets 9, 614–625. 10.2174/138945008785132411 18691009 PMC5783564

[B38] Griesbeck O. Canossa M. Campana G. Gärtner A. Hoener M. C. Nawa H.. (1999). Are there differences between the secretion characteristics of NGF and BDNF? Implications for the modulatory role of neurotrophins in activity-dependent neuronal plasticity. Microsc. Res. Tech. 45, 262–275. 10.1002/(SICI)1097-0029(19990515/01)45:4/5<262::AID-JEMT10>3.0.CO;2-K 10383119

[B39] Groth R. D. Mermelstein P. G. (2003). Brain-derived neurotrophic factor activation of NFAT (nuclear factor of activated T-cells)-dependent transcription: a role for the transcription factor NFATc4 in neurotrophin-mediated gene expression. J. Neurosci. 23, 8125–8134. 10.3410/f.1016012.199381 12954875 PMC6740488

[B40] Guiton M. Gunn-Moore F. J. Stitt T. N. Yancopoulos G. D. Tavare J. M. (1994). Identification of *in vivo* brain-derived neurotrophic factor-stimulated autophosphorylation sites on the TrkB receptor tyrosine kinase by site-directed mutagenesis. J. Biol. Chem. 269, 30370–30377. 7982951

[B41] Haapasalo A. Koponen E. Hoppe E. Wong G. Castrén E. (2001). Truncated trkB.T1 is dominant negative inhibitor of trkB.TK^+^-mediated cell survival. Biochem. Biophys. Res. Commun. 280, 1352–1358. 10.1006/bbrc.2001.4296 11162678

[B42] Hempstead B. L. (2006). Dissecting the diverse actions of pro- and mature neurotrophins. Curr. Alzheimer Res. 3, 19–24. 10.2174/156720506775697061 16472198

[B43] Hofer M. M. Barde Y. A. (1988). Brain-derived neurotrophic factor prevents neuronal death *in vivo*. Nature 331, 261–262. 10.1038/331261a0 3336438

[B44] Hori T. Takai Y. Takahashi T. (1999). Presynaptic mechanism for phorbol ester-induced synaptic potentiation. J. Neurosci. 19, 7262–7267. 10460232 10.1523/JNEUROSCI.19-17-07262.1999 PMC6782531

[B45] Huang S.-H. Zhao L. Sun Z.-P. Li X.-Z. Geng Z. Zhang K.-D.. (2009). Essential role of Hrs in endocytic recycling of full-length TrkB receptor but not its isoform TrkB.T1. J. Biol. Chem. 284, 15126–15136. 10.1074/jbc.m809763200 19351881 PMC2685694

[B46] Je H. S. Yang F. Ji Y. Potluri S. Fu X.-Q. Luo Z.-G.. (2013). ProBDNF and mature BDNF as punishment and reward signals for synapse elimination at mouse neuromuscular junctions. J. Neurosci. 33, 9957–9962. 10.1523/JNEUROSCI.0163-13.2013 23761891 PMC3682390

[B47] Kang B.-S. French O. G. Sando J. J. Hahn C. S. (2000). Activation-dependent degradation of protein kinase Cη. Oncogene 19, 4263–4272. 10.1038/sj.onc.1203779 10980600

[B48] Kermani P. Hempstead B. (2007). Brain-derived neurotrophic factor: a newly described mediator of angiogenesis. Trends Cardiovasc. Med. 17, 140–143. 10.1016/j.tcm.2007.03.002 17482097 PMC2268985

[B49] Kleiman R. J. Tian N. Krizaj D. Hwang T. N. Copenhagen D. R. Reichardt L. F. (2000). BDNF-Induced potentiation of spontaneous twitching in innervated myocytes requires calcium release from intracellular stores. J. Neurophysiol. 84, 472–483. 10899220 10.1152/jn.2000.84.1.472 PMC2710114

[B50] Klein R. Nanduri V. Jing S. Lamballe F. Tapley P. Bryant S.. (1991). The trkB tyrosine protein kinase is a receptor for brain-derived neurotrophic factor and neurotrophin-3. Cell 66, 395–403. 10.1016/0092-8674(91)90628-C 1649702 PMC2710095

[B51] Knipper M. da Penha Berzaghi M. Blöchl A. Breer H. Thoenen H. Lindholm D. (1994). Positive feedback between acetylcholine and the neurotrophins nerve growth factor and brain-derived neurotrophic factor in the rat hippocampus. Eur. J. Neurosci. 6, 668–671. 10.1111/j.1460-9568.1994.tb00312.x 8025717

[B52] Kohara K. Kitamura A. Morishima M. Tsumoto T. (2001). Activity-dependent transfer of brain-derived neurotrophic factor to postsynaptic neurons. Science 291, 2419–2423. 10.1126/science.1057415 11264540

[B53] Koliatsos V. E. Clatterbuck R. E. Winslow J. W. Cayouette M. H. Prices D. L. (1993). Evidence that brain-derived neurotrophic factor is a trophic factor for motor neurons *in vivo*. Neuron 10, 359–367. 10.1016/0896-6273(93)90326-M 8080464

[B54] Kulakowski S. A. Parker S. D. Personius K. E. (2011). Reduced TrkB expression results in precocious age-like changes in neuromuscular structure, neurotransmission and muscle function. J. Appl. Physiol. 111, 844–852. 10.1152/japplphysiol.00070.2011 21737823

[B55] Lanuza M. A. Besalduch N. Garcia N. Sabaté M. Santafé M. M. Tomàs J. (2007). Plastic-embedded semithin cross-sections as a tool for high-resolution immunofluorescence analysis of the neuromuscular junction molecules: specific cellular location of protease-activated receptor-1. J. Neurosci. Res. 85, 748–756. 10.1002/jnr.21192 17265467

[B56] Lanuza M. A. Santafe M. M. Garcia N. Besalduch N. Tomàs M. Obis T.. (2014). Protein kinase C isoforms at the neuromuscular junction: localization and specific roles in neurotransmission and development. J. Anat. 224, 61–73. 10.1111/joa.12106 24102585 PMC3867888

[B57] Lee H. W. Smith L. Pettit G. R. Bingham Smith J. (1996). Dephosphorylation of activated protein kinase C contributes to downregulation by bryostatin. Am. J. Physiol. 271, C304–C311. 8760059 10.1152/ajpcell.1996.271.1.C304

[B58] Li X.-M. Dong X.-P. Luo S.-W. Zhang B. Lee D.-H. Ting A. K. L.. (2008). Retrograde regulation of motoneuron differentiation by muscle β-catenin. Nat. Neurosci. 11, 262–268. 10.1038/nn2053 18278041

[B59] Liem R. S. Brouwer N. Copray J. C. (2001). Ultrastructural localization of intramuscular expression of BDNF mRNA by silver-gold intensified non-radioactive in situ hybridization. Histochem. Cell Biol. 116, 545–551. 10.1007/s00418-001-0349-z 11810196

[B60] Liu G. S. Cohen M. V. Mochly-Rosen D. Downey J. M. (1999). Protein kinase C-epsilon is responsible for the protection of preconditioning in rabbit cardiomyocytes. J. Mol. Cell. Cardiol. 31, 1937–1948. 10.1006/jmcc.1999.1026 10525430

[B61] Lu B. (2003). Pro-region of neurotrophins: role in synaptic modulation. Neuron 39, 735–738. 10.1016/s0896-6273(03)00538-5 12948441

[B62] Lu Z. Liu D. Hornia A. Devonish W. Pagano M. Foster D. A. (1998). Activation of protein kinase C triggers its ubiquitination and degradation. Mol. Cell. Biol. 18, 839–845. 10.1128/mcb.18.2.839 9447980 PMC108795

[B63] Luo B. Huang J. Lu L. Hu X. Luo Z. Li M. (2014). Electrically induced brain-derived neurotrophic factor release from schwann cells. J. Neurosci. Res. 92, 893–903. 10.1002/jnr.23365 24753179

[B111] Mantilla C. B. Seven Y. B. Sieck G. C. (2014). Convergence of pattern generator outputs on a common mechanism of diaphragm motor unit recruitment. Prog. Brain Res. 209, 309–329. 10.1016/B978-0-444-63274-6.00016-3 24746055 PMC4154308

[B64] Mantilla C. B. Zhan W.-Z. Sieck G. C. (2004). Neurotrophins improve neuromuscular transmission in the adult rat diaphragm. Muscle Nerve 29, 381–386. 10.1002/mus.10558 14981737

[B65] Matthews V. B. Aström M.-B. Chan M. H. S. Bruce C. R. Krabbe K. S. Prelovsek O.. (2009). Brain-derived neurotrophic factor is produced by skeletal muscle cells in response to contraction and enhances fat oxidation via activation of AMP-activated protein kinase. Diabetologia 52, 1409–1418. 10.1007/s00125-009-1364-1 19387610

[B66] McLachlan E. M. Martin A. R. (1981). Non-linear summation of end-plate potentials in the frog and mouse. J. Physiol. 311, 307–324. 10.1113/jphysiol.1981.sp013586 6267255 PMC1275411

[B67] Meyer-Franke A. Wilkinson G. A. Kruttgen A. Hu M. Munro E. Hanson M. G.. (1998). Depolarization and cAMP elevation rapidly recruit TrkB to the plasma membrane of CNS neurons. Neuron 21, 681–693. 10.1016/s0896-6273(00)80586-3 9808456 PMC2693071

[B68] Middlemas D. S. Lindberg R. A. Hunter T. (1991). TrkB, a neural receptor protein-tyrosine kinase: evidence for a full-length and two truncated receptors. Mol. Cell. Biol. 11, 143–153. 10.1128/mcb.11.1.143 1846020 PMC359604

[B69] Middlemas D. S. Meisenhelder J. Hunter T. (1994). Identification of TrkB autophosphorylation sites and evidence that phospholipase C-gamma 1 is a substrate of the TrkB receptor. J. Biol. Chem. 269, 5458–5466. 8106527

[B70] Mowla S. J. Pareek S. Farhadi H. F. Petrecca K. Fawcett J. P. Seidah N. G. (1999). Differential sorting of nerve growth factor and brain-derived neurotrophic factor in hippocampal neurons. J. Neurosci. 19, 2069–2080. 10066260 10.1523/JNEUROSCI.19-06-02069.1999 PMC6782557

[B71] Neeper S. A. Góauctemez-Pinilla F. Choi J. Cotman C. (1995). Exercise and brain neurotrophins. Nature 373:109. 10.1038/373109a0 7816089

[B72] Newton A. C. (2003). Regulation of the ABC kinases by phosphorylation: protein kinase C as a paradigm. Biochem. J. 370, 361–371. 10.1042/bj20021626 12495431 PMC1223206

[B73] Numann R. Hauschka S. D. Catterall W. A. Scheuer T. (1994). Modulation of skeletal muscle sodium channels in a satellite cell line by protein kinase C. J. Neurosci. 14, 4226–4236. 8027774 10.1523/JNEUROSCI.14-07-04226.1994 PMC6577030

[B74] Obermeier A. Halfter H. Wiesmüller K. H. Jung G. Schlessinger J. Ullrich A. (1993). Tyrosine 785 is a major determinant of Trk—substrate interaction. EMBO J. 12, 933–941. 8384556 10.1002/j.1460-2075.1993.tb05734.x PMC413294

[B75] Obis T. Besalduch N. Hurtado E. Nadal L. Santafe M. M. Garcia N.. (2015a). The novel protein kinase C epsilon isoform at the adult neuromuscular synapse: location, regulation by synaptic activity-dependent muscle contraction through trkB signaling and coupling to ACh release. Mol. Brain 8:8. 10.1186/s13041-015-0098-x 25761522 PMC4348107

[B76] Obis T. Hurtado E. Nadal L. Tomàs M. Priego M. Simon A.. (2015b). The novel protein kinase C epsilon isoform modulates acetylcholine release in the rat neuromuscular junction. Mol. Brain 8:80. 10.1186/s13041-015-0171-5 26625935 PMC4665914

[B77] Ozek C. Kanoski S. E. Zhang Z. Y. Grill H. J. Bence K. K. (2014). Protein-tyrosine phosphatase 1B (PTP1B) is a novel regulator of central brain-derived neurotrophic factor and tropomyosin receptor kinase B (TrkB) signaling. J. Biol. Chem. 289, 31682–31692. 10.1074/jbc.M114.603621 25288805 PMC4231649

[B78] Patterson S. L. Pittenger C. Morozov A. Martin K. C. Scanlin H. Drake C.. (2001). Some forms of cAMP-mediated long-lasting potentiation are associated with release of BDNF and nuclear translocation of phospho-MAP kinase. Neuron 32, 123–140. 10.1016/S0896-6273(01)00443-3 11604144

[B79] Pfaffl M. W. Horgan G. W. Dempfle L. (2002). Relative expression software tool (REST© ) for group-wise comparison and statistical analysis of relative expression results in real-time PCR. Nucleic Acids Res. 30:e36. 10.1093/nar/30.9.e36 11972351 PMC113859

[B80] Reichardt L. F. (2006). Neurotrophin-regulated signaling pathways. Philos. Trans. R Soc. Lond. B Biol. Sci. 361, 1545–1564. 10.1098/rstb.2006.1894 16939974 PMC1664664

[B81] Rosato Siri M. D. Uchitel O. D. (1999). Calcium channels coupled to neurotransmitter release at neonatal rat neuromuscular junctions. J. Physiol. 514, 533–540. 10.1111/j.1469-7793.1999.533ae.x 9852333 PMC2269071

[B82] Rose C. R. Blum R. Pichler B. Lepier A. Kafitz K. W. Konnerth A. (2003). Truncated TrkB-T1 mediates neurotrophin-evoked calcium signaling in glia cells. Nature 426, 74–78. 10.1038/nature01983 14603320

[B83] Rusanescu G. Yang W. Bai A. Neel B. G. Feig L. A. (2005). Tyrosine phosphatase SHP-2 is a mediator of activity-dependent neuronal excitotoxicity. EMBO J. 24, 305–314. 10.1038/sj.emboj.7600522 15650750 PMC545812

[B84] Sagot Y. Rossé T. Vejsada R. Perrelet D. Kato A. C. (1998). Differential effects of neurotrophic factors on motoneuron retrograde labeling in a murine model of motoneuron disease. J. Neurosci. 18, 1132–1141. 9437033 10.1523/JNEUROSCI.18-03-01132.1998 PMC6792750

[B85] Santafé M. M. Garcia N. Tomàs M. Obis T. Lanuza M. A. Besalduch N.. (2014). The interaction between tropomyosin-related kinase B receptors and serine kinases modulates acetylcholine release in adult neuromuscular junctions. Neurosci. Lett. 561, 171–175. 10.1016/j.neulet.2013.12.073 24406154

[B86] Santafé M. M. Lanuza M. A. Garcia N. Tomàs J. (2005). Calcium inflow-dependent protein kinase C activity is involved in the modulation of transmitter release in the neuromuscular junction of the adult rat. Synapse 57, 76–84. 10.1002/syn.20159 15906390

[B87] Santafé M. M. Lanuza M. A. Garcia N. Tomàs J. (2006). Muscarinic autoreceptors modulate transmitter release through protein kinase C and protein kinase A in the rat motor nerve terminal. Eur. J. Neurosci. 23, 2048–2056. 10.1111/j.1460-9568.2006.04753.x 16630052

[B88] Segal R. A. Bhattacharyya A. Rua L. A. Alberta J. A. Stephens R. M. Kaplan D. R.. (1996). Differential utilization of Trk autophosphorylation sites. J. Biol. Chem. 271, 20175–20181. 10.1074/jbc.271.33.20175 8702742

[B110] Seven Y. B. Mantilla C. B. Sieck G. C. (2014). Recruitment of rat diaphragm motor units across motor behaviors with different levels of diaphragm activation. J. Appl. Physiol. 117, 1308–1316. 10.1152/japplphysiol.01395.2013 25257864 PMC4254843

[B89] Stoilov P. Castren E. Stamm S. (2002). Analysis of the human TrkB gene genomic organization reveals novel TrkB isoforms, unusual gene length and splicing mechanism. Biochem. Biophys. Res. Commun. 290, 1054–1065. 10.1006/bbrc.2001.6301 11798182

[B90] Suen P.-C. Wu K. Levine E. S. Mount H. T. J. Xu J.-L. Lin S.-Y.. (1997). Brain-derived neurotrophic factor rapidly enhances phosphorylation of the postsynaptic N-methyl-D-aspartate receptor subunit 1 (Postsynaptic receptorneurotrophinsynaptic plasticity). Proc. Natl. Acad. Sci. U S A 94, 8191–8195. 9223337 10.1073/pnas.94.15.8191 PMC21579

[B91] Takei N. Kawamura M. Hara K. Yonezawa K. Nawa H. (2001). Brain-derived neurotrophic factor enhances neuronal translation by activating multiple initiation processes. comparison with the effects of insulin. J. Biol. Chem. 276, 42818–42825. 10.1074/jbc.m103237200 11551908

[B92] Takei N. Numakawa T. Kozakit S. Sakai N. Endo Y. Takahashi M.. (1998). Brain-derived neurotrophic factor induces rapid and transient release of glutamate through the non-exocytotic pathway from cortical neurons. J. Biol. Chem. 273, 27620–27624. 10.1074/jbc.273.42.27620 9765296

[B93] Todd K. J. Auld D. S. Robitaille R. (2007). Neurotrophins modulate neuron-glia interactions at a vertebrate synapse. Eur. J. Neurosci. 25, 1287–1296. 10.1111/j.1460-9568.2007.05385.x 17355253

[B108] Tremblay F. Brûlé S. Hee Um S. Li Y. Masuda K. Roden M.. (2007). Identification of IRS-1 Ser-1101 as a target of S6K1 in nutrient- and obesity-induced insulin resistance. Proc. Natl. Acad. Sci. U S A 104, 14056–14061. 10.1073/pnas.0706517104 17709744 PMC1950339

[B94] Urbano F. J. Piedras-Rentería E. S. Jun K. Shin H.-S. Uchitel O. D. Tsien R. W. (2003). Altered properties of quantal neurotransmitter release at endplates of mice lacking P/Q-type Ca^2+^ channels. Proc. Natl. Acad. Sci. U S A 100, 3491–3496. 10.1073/pnas.0437991100 12624181 PMC152320

[B95] van Praag H. Kempermann G. Gage F. H. (1999). Running increases cell proliferation and neurogenesis in the adult mouse dentate gyrus. Nat. Neurosci. 2, 266–270. 10.1038/6368 10195220

[B96] Vaynman S. S. Ying Z. Yin D. Gomez-Pinilla F. (2006). Exercise differentially regulates synaptic proteins associated to the function of BDNF. Brain Res. 1070, 124–130. 10.1016/j.brainres.2005.11.062 16413508

[B97] Wang T. Xie K. Lu B. (1995). Neurotrophins promote maturation of developing neuromuscular synapses. J. Neurosci. 15, 4796–4805. 7623111 10.1523/JNEUROSCI.15-07-04796.1995 PMC6577890

[B98] West J. W. Numann R. Murphy B. J. Scheuer T. Catterall W. A. (1991). A phosphorylation site in the Na^+^ channel required for modulation by protein kinase C. Science 254, 866–868. 10.1126/science.1658937 1658937

[B99] Wong J. Garner B. (2012). Evidence that truncated TrkB isoform, TrkB-Shc can regulate phosphorylated TrkB protein levels. Biochem. Biophys. Res. Commun. 420, 331–335. 10.1016/j.bbrc.2012.02.159 22425982

[B100] Woo N. H. Teng H. K. Siao C.-J. Chiaruttini C. Pang P. T. Milner T. A.. (2005). Activation of p75NTR by proBDNF facilitates hippocampal long-term depression. Nat. Neurosci. 8, 1069–1077. 10.1038/nn1510 16025106

[B101] Wu H. Xiong W. C. Mei L. (2010). To build a synapse: signaling pathways in neuromuscular junction assembly. Development 137, 1017–1033. 10.1242/dev.038711 20215342 PMC2835321

[B102] Yan Q. Elliott J. Snider W. D. (1992). Brain-derived neurotrophic factor rescues spinal motor neurons from axotomy-induced cell death. Nature 360, 753–755. 10.1038/360753a0 1281520

[B103] Yang J. Siao C.-J. Nagappan G. Marinic T. Jing D. McGrath K.. (2009). Neuronal release of proBDNF. Nat. Neurosci. 12, 113–115. 10.1038/nn.2244 19136973 PMC2737352

[B104] Yang X. Arber S. William C. Li L. Tanabe Y. Jessell T. M.. (2001). Patterning of muscle acetylcholine receptor gene expression in the absence of motor innervation. Neuron 30, 399–410. 10.1016/S0896-6273(01)00287-2 11395002

[B109] Yung H. W. Charnock-Jones D. S. Burton G. J. (2011). Regulation of AKT phosphorylation at Ser473 and Thr308 by endoplasmic reticulum stress modulates substrate specificity in a severity dependent manner. PLoS One 6:e17894. 10.1371/journal.pone.0017894 21445305 PMC3061875

[B105] Zhang Y. Ying J. Jiang D. Chang Z. Li H. Zhang G.. (2015). Urotensin-II receptor stimulation of cardiac L-type Ca^2+^ channels requires the βγ subunits of Gi/o-protein and phosphatidylinositol 3-kinase-dependent protein kinase C β1 isoform. J. Biol. Chem. 290, 8644–8655. 10.1074/jbc.m114.615021 25678708 PMC4375513

[B106] Zheng Z. Sabirzhanov B. Keifer J. (2010). Oligomeric amyloid-beta inhibits the proteolytic conversion of brain-derived neurotrophic factor (BDNF), AMPA receptor trafficking and classical conditioning. J. Biol. Chem. 285, 34708–34717. 10.1074/jbc.M110.150821 20807770 PMC2966086

[B107] Zoladz J. A. Pilc A. (2010). The effect of physical activity on the brain derived neurotrophic factor: from animal to human studies. J. Physiol. Pharmacol. 61, 533–541. 21081796

